# SLC7A9 suppression increases chemosensitivity by inducing ferroptosis via the inhibition of cystine transport in gastric cancer

**DOI:** 10.1016/j.ebiom.2024.105375

**Published:** 2024-10-21

**Authors:** Haoran Feng, Junxian Yu, Zhuoqing Xu, Qingqing Sang, Fangyuan Li, Mengdi Chen, Yunqin Chen, Beiqin Yu, Nan Zhu, Jiazeng Xia, Changyu He, Junyi Hou, Xiongyan Wu, Chao Yan, Zhenggang Zhu, Liping Su, Jianfang Li, Wentao Dai, Yuan-Yuan Li, Bingya Liu

**Affiliations:** a Department of General Surgery, Shanghai Key Laboratory of Gastric Neoplasms, Shanghai Institute of Digestive Surgery, Ruijin Hospital, Shanghai Jiao Tong University School of Medicine, Shanghai 200025, China; b Shanghai-MOST Key Laboratory of Health and Disease Genomics & NHC Key Laboratory of Reproduction Regulation, Shanghai Institute for Biomedical and Pharmaceutical Technologies, Fudan University, Shanghai 200080, China; c Department of General Surgery, Jiangnan University Medical Center, Wuxi 200240, China

**Keywords:** Gastric cancer, Ferroptosis, SLC7A9, Glutathione metabolism, GPX4

## Abstract

**Background:**

SLC7A9 is responsible for the exchange of dibasic amino acids and cystine (influx) for neutral amino acids (efflux). Cystine/cysteine transport is related to ferroptosis.

**Methods:**

Sanger sequencing detected *TP53* status of cancer cells. Transcriptomic sequencing and untargeted metabolome profiling were used to identify differentially expressed genes and metabolites, respectively, upon SLC7A9 overexpression. CCK8, cell clonality, and EdU assays were used to observe cell proliferation. Cystine probes, glutathione (GSH) probes, and lipid ROS probes were used to examine cystine, GSH, and lipid ROS levels. ^13^C metabolic flow assays were used to monitor cellular cystine and GSH metabolism. Patient-derived organoids (PDO), immunocompetent MFC mice allograft models and patient-derived xenograft (PDX) models were used to evaluate SLC7A9 impact on chemotherapeutic response and to observe therapeutic effect of SLC7A9 knockdown.

**Findings:**

Elevated SLC7A9 expression levels in gastric cancer cells were attributed to p53 loss. SLC7A9 knockdown suppressed the proliferation and increased the chemotherapy sensitivity of the cells. Chemotherapy was more effective in PDX and immunocompetent mice models upon SLC7A9 knockdown. Differentially expressed genes and metabolites between the SLC7A9 overexpression and control groups were associated with ferroptosis and GSH metabolism. SLC7A9 knockdown reduced cystine transport into cells, hampered intracellular cystine and GSH metabolic flow, decreased GSH synthesis, and increased lipid ROS levels in gastric cancer cells. Erastin was more effective at inducing ferroptosis in PDO and PDX models upon SLC7A9 knockdown.

**Interpretation:**

SLC7A9 promotes gastric cancer progression by acting as a suppressor of ferroptosis, independent of SLC7A11, which is negatively regulated by p53.

**Funding:**

This work was supported by 10.13039/501100001809National Natural Science Foundation of China, Innovation Promotion Program of NHC and Shanghai Key Labs SIBPT, and Shanghai Academy of Science & Technology.

**Research in context**

**Evidence before this study**

Solute carrier family 7 member 9 (SLC7A9) is responsible for the exchange of dibasic amino acids and cystine (influx) for neutral amino acids (efflux). The transport of cystine/cysteine is related to ferroptosis. Previous studies have described the role of ferroptosis in the clearance of tumour cells that lack access to critical nutrients in their microenvironment. Targeted therapies are employed in the treatment of only a small proportion of patients. The development of new therapeutic strategies for gastric cancer, based on ferroptosis, remains challenging.

**Added value of this study**

SLC7A9 expression is upregulated in TP53-deficient gastric cancer. SLC7A9 promotes gastric cancer progression by inhibiting ferroptosis via regulation of cystine and glutamate transport, glutathione metabolism, and redox equilibrium. SLC7A9 upregulation induces chemoresistance by inhibiting ferroptosis, independent of SLC7A11.

**Implications of all the available evidence**

These findings indicate that SLC7A9 acts as a ferroptosis inhibitor, independent of SLC7A11. Therefore, therapeutic interventions targeting SLC7A9 are promising for sensitising gastric cancer cells to ferroptosis and chemotherapy treatment, and ultimately, improving the prognosis of patients with gastric cancer.

## Introduction

Gastric cancer is one of the five most commonly diagnosed malignancies, with more than one million new cases diagnosed annually. It was the fourth leading cause of cancer-related deaths globally in 2020.^1^ Gastric cancer is highly heterogeneous and has a poor prognosis. Although early-stage gastric cancer can be effectively treated via surgical resection, most patients are diagnosed at advanced stages. For these patients, surgical treatment combined with adjuvant chemotherapy remains the primary treatment.^2^ In addition to adjuvant chemotherapy, targeted therapies, including human epidermal growth factor receptor 2 and vascular endothelial growth factor receptor inhibitors are also used.^2^^,^^3^ However, these targeted therapies have been employed in the treatment of only a small proportion of patients. Therefore, the development of new therapeutic strategies for gastric cancer is required.

Most therapies exert their antitumour effects via regulated cell death (RCD) pathways, such as apoptosis, necroptosis, pyroptosis, and ferroptosis. Ferroptosis is a form of oxidative RCD with unique morphological, biochemical, and genetic characteristics.^4^ Previous studies have described the role of ferroptosis in the clearance of tumour cells that lack access to critical nutrients in their microenvironment.^5^ Specifically, cellular stress resulting from aberrant metabolic and biochemical processes can lead to the continuous accumulation of lipid hydroperoxides and trigger iron-dependent ferroptosis.^5^

The hallmarks of ferroptosis include three essential biological processes: 1) loss of lipid peroxide repair capacity by phospholipid hydroperoxidase glutathione peroxidase 4 (GPX4), 2) the accumulation of redox-active iron, and 3) the oxidation of polyunsaturated fatty acid (PUFA)-containing phospholipids.^6^ First, abundant PUFAs increase the degree of lipid peroxidation, and hence, determine cellular sensitivity to ferroptosis.^5^^,^^7^^,^^8^ Second, iron is required for the accumulation of lipid peroxides and it functions as a catalyst of ferroptosis.^9^ Finally, the inactivation of GPX4, an enzyme that converts toxic lipid hydroperoxides into nontoxic lipid alcohols, allows the hydroperoxyl derivatives of PUFAs to induce ferroptotic cell death.^5^^,^^10^, ^11^, ^12^ GPX4 activity is regulated by the cystine/glutamate antiporter system X_C_^−^. This system consists of solute carrier family 7 member 11 (SLC7A11), which plays a canonical role in cystine import and glutathione (GSH) production, and can maintain the activity of GPX4 and inhibit ferroptosis.^10^^,^^13^^,^^14^ Furthermore, p53, the classical cancer suppressor, also regulates ferroptosis in cancer cells.^4^^,^^15^ Previous studies have found that p53 activation can significantly enhance intracellular oxidation levels by inhibiting SLC7A11 expression, ultimately inducing ferroptosis.^4^^,^^15^ Hence, amino acid transport and GSH metabolism are closely linked to the regulation of ferroptosis.^5^^,^^9^^,^^16^

SLC7A11 plays a key role in ferroptosis and cancer development.^17^ SLC7A9, another member of solute carrier family 7, is the light subunit of a transmembrane amino acid transporter,^18^ which exchanges dibasic amino acids and cystine (influx) for neutral amino acids (efflux) and functions in the reabsorption of cystine in the renal tubules.^18^^,^^19^ Many studies have focused on the relationship between SLC7A9 and chronic kidney disease, and SLC7A9 mutations have been found to lead to various types of cystinuria.^18^, ^19^, ^20^ Additionally, researchers have identified a relationship between SLC7A9 expression levels and intracellular GSH levels.^21^ These findings indicate that SLC7A9 may play a role in the regulation of ferroptosis. In our previous differential regulation network (DRN) analysis of gastric cancer, SLC7A9 was identified as the most significant differentially regulated gene (DRG) between gastric cancer and adjacent tissues.^22^

Here, we demonstrate that SLC7A9 expression is upregulated in *TP53*-deficient gastric cancer, and that upregulation of SLC7A9 induces chemoresistance by inhibiting ferroptosis, independent of SLC7A11, suggesting that SLC7A9 acts as a ferroptosis suppressor.

## Methods

### Antibodies and reagents

The antibody against GAPDH (ab8245, RRID: AB_2107448) was purchased from Abcam (Cambridge, UK). The antibody against ki67 (GB111499, RRID: AB_2927572) was purchased from Servicebio (Wuhan, China). Antibodies against GPX4 (52455, RRID: AB_2924984) and p53 (48818, RRID: AB_2713958) were purchased from Cell Signalling Technology (Boston, MA, USA). The 5-ethynyl-2′-deoxyuridine (EdU) assay kit (C0075) was obtained from Beyotime Biotechnology (Shanghai, China). The cystine enzyme-linked immunosorbent assay (ELISA, H96-3402E) and GSH assay (U96-3510E) kits were obtained from YOBIBIO (Shanghai, China). The chromatin immunoprecipitation (ChIP) assay kit (26157) was obtained from Thermo Fisher Scientific (Waltham, MA, USA). The Cell Counting Kit-8 assay kit (MA0218) was obtained from Meilunbio (Dalian, China). Erastin (T1765), oxaliplatin (PT, T0164), and 5-fluorouracil (5-FU, T0984) were obtained from TargetMol (Boston, MA, USA). Antibodies against SLC7A11 (A2413, RRID: AB_2863004) and SLC7A9 (A12848, RRID: AB_2759689) were obtained from Abclonal (Wuhan, China). The cystine-fluorescein isothiocyanate (FITC) probes were purchased from Sigma (SCT047; St Louis, MO, USA).

### Bioinformatics analysis

p53 binding sites in the *SLC7A9* promoter region were predicted using Jaspar (https://jaspar.genereg.net/). Statistical analyses were performed using R software (version 4.0.2). The endpoint of the Kaplan–Meier analysis was overall survival, and the log-rank test or hazard ratio test was used to determine statistical significance.

### Cell culture

Human gastric cancer AGS cells (RRID:CVCL_0139), SGC7901 cells (RRID:CVCL_0520), HGC27 cells (RRID:CVCL_1279), NCI–N87 cells (RRID:CVCL_1603), HS-746T cells (RRID:CVCL_0333), MKN45 cells (RRID:CVCL_0434), MGC803 cells (RRID:CVCL_5334), and FU97 cells (RRID:CVCL_2908) and human immortalised stomach epithelial cell line GES1 (RRID:CVCL_EQ22) were purchased from the American Type Culture Collection (Manassas, VA, USA) and cultured in RPMI-1640 medium (Meilunbio) containing 10% foetal bovine serum (Gibco, Grand Island, NY, USA), 100 U/mL of penicillin, and 100 μg/mL of streptomycin. Cells were maintained in a humidified culture incubator at 37 °C under conditions containing 5% CO_2_.

### Cell viability assay

Cell viability was evaluated using CCK8. AGS/vector cells, AGS/oeSLC7A9 (SLC7A9-overexpressing AGS cells) cells, FU97/NC cells, and FU97/shSLC7A9 cells were seeded in 96-well plates containing 200 μL of RPMI-1640 medium at a density of 2 × 10^3^ cells/well. When monitoring is needed, the original medium was discarded, and 100 μL of RPMI-1640 medium with 10% CCK8 solution was added to each well. The cells were then incubated at 37 °C for 2 h. Absorbance was measured at 450 nm at the specified time using a microplate reader.

### ChIP assays

Briefly, the cells were cross-linked with formalin. After ultrasonication, chromosomes were broken into small fragments. The lysates were incubated with primary antibodies at 4 °C overnight. A total of 5 mg of antibodies was used in 100 μL of lysate originating from 1 × 10^6^ cells for each experiment. Magnetic beads were added to the lysate to capture the antibody-protein-DNA complexes. After centrifugation and washing, bound DNA was obtained by reverse crosslinking and purification. The DNA was quantified using quantitative polymerase chain reaction (qPCR). ChIP data were calculated as percentages relative to the input DNA using the following equation: 2^(input Ct-target Ct)^. The primers used for qPCR were as follows: first: forward, 5′-GTCAGGAGTTCAAGACCAACCTG, reverse, 5′-GCACGATCTCAGCTCACTGCAAC; second: forward, 5′-TCCTGGCCTTGTGTTGCCTGTC, reverse, 5′-AGCAGGCCTGAGGCAATGCAG.

### Colony-formation assay

A total of 1 × 10^3^ cells were seeded in a six-well plate. After incubation for 2 weeks, the cell colonies were washed with PBS, fixed with 4% paraformaldehyde, stained with 0.5% crystal violet for 2 h, and dried. The colonies were photographed using an inverted microscope.

### Evaluation of cystine concentration

The cystine concentration was detected using a cystine ELISA kit according to the manufacturer’s instructions.

### Dual-luciferase reporter assay

Full-length sequences of the *SLC7A9* promoter were synthesised and incorporated into the pGL3-basic plasmid. HEK293T cells were seeded in 24-well plates and transfected with pcDNA3.1- *TP53* or pcDNA 3.1 plasmids, pGL3-Basic-SLC7A9, and pRL-TK plasmids using Lipofectamine 3000 (Invitrogen, Carlsbad, CA, USA). Three days after transfection, a dual-luciferase reporter assay was performed using the Dual-Luciferase Reporter Assay System (Meilunbio) according to the manufacturer’s instructions.

### EdU assay

An EdU-incorporation assay kit was used to determine the cell proliferation rate. Briefly, RPMI-1640 medium was added to six-well plates seeded with gastric cancer cells. After treatment, the cells were incubated with an EdU working solution for 2 h and fixed with 4% paraformaldehyde. Subsequently, 555 Azide was added to the cells treated with 0.5% Triton X100, followed by incubation for 30 min. The cells were then stained with Hoechst 33342. Finally, the percentage of fluorescence-positive cells was calculated to assess the proliferation rate.

### Flow cytometry

To detect cystine-FITC in cells, flow cytometry was performed as previously described.^23^ Before cystine-FITC usage, the cells were subjected to PBS starvation for 40 min. They were then incubated with 5 μM cystine-FITC for 40 min before flow cytometric analysis.

### Gene set enrichment analysis

Gene set enrichment analysis (GSEA) was performed to determine the significantly enriched pathways based on data from the MSigDB database (c2.cp.kegg.v7.2.symbols.gmt gene sets). The top 20 positively correlated pathways were plotted using the R package ggplot2.

### GSH and glutathione disulfide detection

Intracellular GSH and glutathione disulfide (GSSG) levels were measured using specific assay kits, according to the manufacturer’s instructions.

### Haematoxylin and eosin staining

The tumours collected from mice were fixed with 4% paraformaldehyde. Paraffin-embedded sections were stained with haematoxylin and eosin (HE) and then viewed and photographed under a microscope.

### Identification of differential gene expression

DESeq^24^ and Q-values were used to evaluate differential gene expression between queen and worker honeybees. Subsequently, differences in gene abundance between samples were calculated based on the ratio of fragments per kilobase of exon per million fragments mapped (FPKM) values. The false-discovery rate (FDR) control method was used to identify the *p*-value threshold for multiple tests, to compute statistical significance. Only genes with an absolute log2 ratio ≥2 and an FDR significance score <0.01 were used for subsequent analysis.

### Immunofluorescence assay

Paraffin sections were treated with xylene to remove the paraffin. They were then dehydrated using an ethanol gradient. For antigen retrieval, the sections were covered with 0.01 mol/L sodium citrate buffer (pH 6.0) and heated at 100 °C for 15 min. After blocking with 5% bovine serum albumin for 30 min, the sections were incubated overnight at 4 °C with primary antibodies against SLC7A9 and ki67. The sections were then incubated with Alexa Fluor™ 488– or Alexa Fluor™ 555–conjugated secondary antibodies (Abcam) at 37 °C for 1 h. 4′6-Diamidino-2-phenylindole was used for nuclear staining. Laser-scanning confocal microscopy (LSM510; Zeiss, Oberkochen, Germany) was used to visualise the results.

### Immunohistochemistry

Tissues were fixed in 4% paraformaldehyde and embedded in paraffin. The paraffin-embedded tissue blocks were dewaxed, hydrated, and subjected to antigen retrieval. After washing, the slides were treated with 3% hydrogen peroxide for 15 min, washed, and blocked with bovine serum albumin for 15 min at room temperature. Subsequently, anti-SLC7A9 (1:1200), anti-ki67 (1:400), or anti-GPX4 (1:800) antibodies were added to the sections and incubated at 4 °C overnight. Streptavidin peroxidase was used for signal detection, followed by staining with diaminobenzidine and counterstaining with haematoxylin. The sections were observed and photographed under a light microscope. All slides were scored by two independent observers in a blinded manner.

### Plasmid construction and RNA interference

The lentiviral expressing shRNA targeting the sequence of SLC7A9 gene (5′- CCTCAAATCGTTGTGAAAT-3′) were synthesized and cloned into the ZV102 shRNA lentivector (U6-shRNA-PGK-PURO; Bioegene). Full-length human SLC7A9 cDNA were synthesized and subcloned into the FV026 overexpression lentivector (CMV-MCS-IRES-PURO; Bioegene). SLC7A11 siRNA (sense 5′-CCUGUCACUAUUUGGAGCUUUdTdT-3’, antisense 5′-AAAGCUCCAAAUAGUGACAGGdTdT-3′), and P53 siRNA (sense 5′-GCGCACAGAGGAAGAGAAUdTdT-3′antisense 5′AUUCUCUUCCUCUGUGCGCdTdT-3′) were synthesized by BioeGene Co., Ltd. (Shanghai, China).

### Lentiviral transduction

Transfection and cell selection were performed as previously described.^23^ The expression efficiency was evaluated using qPCR and western blotting analyses.

### Measurement of lipid ROS levels

ROS levels were measured using a lipid peroxidation assay. Lipid peroxidation was measured using C11-BODIPY (581/591). Cells in six-well plates were incubated with 10 μM C11-BODIPY (581/591) at 37 °C for 40 min. The cells were then harvested and resuspended for flow cytometric analysis.

### Metabolomics analysis

Samples were collected from pretreated cells. After centrifugation, ultrasonication, and extraction, the samples were analysed using a Waters Acquity I-class Plus Xevo G2-XS QTof system. The following chromatographic column was used: Acquity UPLC HSS T3 (1.8 μm; 2.1 ∗ 100 nm). Raw data were collected using MassLynx V4.2 and analysed using Progenesis QI with the online METLIN metabolite database (https://metlin.scripps.edu/).

### Patients and specimens

Tumour and adjacent non-tumour tissue specimens were collected from 69 patients with gastric cancer. Patients who underwent preoperative treatments, such as radiation or chemotherapy, were excluded from the study. All procedures involving humans were approved by the Ethics Committee of the Shanghai Ruijin Hospital, Shanghai Jiao Tong University School of Medicine, Shanghai, China (Approval No. 2017-0003). All samples were obtained with informed consent from the patients. We did not specifically focus on sex differences in this study. The sex information was self-reported by the patients and corresponded to their biological sex.

### qPCR analysis

Cells were lysed using TRIzol reagent (Solarbio, Beijing, China), and total RNA was extracted with chloroform and isopropyl alcohol. cDNA was synthesised using a reverse transcription reagent kit (Vazyme Biotech, Nanjing, China) according to the manufacturer’s protocol. A SYBR Green Master Mix Kit (Vazyme Biotech) was used for the relative quantification of RNA levels according to the manufacturer’s instructions. *GAPDH* was selected as an internal control. The sequences of the primers were as follows: *GAPDH:* forward, 5′-GCACCGTCAAGGCTGAGAAC and reverse, 5′-ATGGTGGTGAAGACGCCAGT; SLC7A9: forward, 5′-TGGGCACCATCATTGGCTC and reverse, 5′-GGCCTCCATCAGGTAGGGAT. The expression levels were normalised to those of the internal control and determined using the 2^−ΔΔCT^ method.

### RNA extraction, library construction, and RNA sequencing

Total RNA was extracted using TRIzol Reagent (Life Technologies, Carlsbad, CA, USA). RNA integrity and concentration were assessed using an Agilent 2100 Bioanalyzer (Agilent Technologies, Inc., Santa Clara, CA, USA). mRNA was isolated using the NEBNext Poly (A) mRNA Magnetic Isolation Module (E7490; New England Biolabs, Ipswich, MA, USA). A cDNA library was constructed using the NEBNext Ultra RNA Library Prep Kit for Illumina (E7530, New England Biolabs) and NEBNext Multiplex Oligos for Illumina (E7500, New England Biolabs) according to the manufacturer’s instructions. Briefly, enriched mRNA was fragmented into approximately 200 nt RNA inserts, which were used to synthesise first- and second-strand cDNA. Double-stranded cDNA was subjected to end-repair, A-tailing and adaptor ligation. Suitable fragments were isolated using Agencourt AMPure XP beads (Beckman Coulter, Inc., Brea, CA, USA) and enriched by PCR amplification. Finally, the constructed cDNA libraries were sequenced using the HiSeq™ sequencing platform (Illumina, San Diego, CA, USA).

### Sanger sequencing

The entire coding region of the *TP53* gene (exon 4 to exon 8) from gastric cancer cell lines was amplified by PCR. The PCR fragments were sequenced using Sanger sequencing. The PCR and sequencing primers are listed in Supplementary Table S1. Sequence chromatograms were visually inspected using DNA Dynamo Sequence Analysis Software (Blue Tractor Software, Wales). All mutations were confirmed by independent PCR and sequencing analyses. We only considered nucleotide variations as mutations if they were present in the tumour samples and not in the normal tissue samples. For non-silent single nucleotide substitutions, sorting intolerant from tolerant analysis was performed to predict whether amino acid substitutions affected protein function.

### Transcriptome analysis using reference-genome-based read mapping

Low-quality reads, such as those with only adaptors, unknown nucleotides >5%, or Q20 < 20% (percentage of sequences with sequencing error rates <1%), were removed using PerlScript. Clean reads that were filtered from the raw reads and mapped to the honeybee (*Apis mellifera*) genome (OGSv3.2) using Tophat2^25^ software. The alignment records in BAM/SAM format were further examined to remove potential duplicates. Gene expression levels were estimated using FPKM values employing Cufflinks software.^26^

### Western blotting

Briefly, cells were lysed in radioimmunoprecipitation assay buffer on ice after washing with PBS. The protein concentration was quantified using a BCA Protein Assay Kit (Thermo Fisher Scientific). Subsequently, equal amounts of protein were separated by sodium dodecyl sulphate–polyacrylamide gel electrophoresis and transferred to polyvinylidene difluoride membranes. The membranes were blocked with 5% skim milk for 1 h and incubated with primary antibodies at 4 °C overnight. After washing thrice with Tris-buffered saline with 0.1% Tween 20, the membranes were incubated with secondary antibodies at room temperature for 1 h and washed again. Blots were visualised using a chemiluminescence detection kit (ECL-PLUS).

### Targeted metabolomics analysis of amino acids (^13^C metabolic flow assay)

^13^C-labelled metabolites were added to the cell culture medium. After culturing for 0.5 h, the cells were digested and stored at −80 °C. Metabolite extraction methods was carried out according to the chemical characteristics of the targeted metabolites. The extract samples were purified by OE Biotech Co., Ltd. (Shanghai, China) according to their standard protocols. Briefly, the standard samples were [^13^C2]-L-cystine and L-glutathione reduced-^13^C. The concentrations of [^13^C2]-L-cystine and L-glutathione reduced-^13^C were both 20 μg/mL. Ultra-high-performance liquid chromatography–electrospray ionisation–tandem mass spectrometry was used for qualitative and quantitative analyses of three substances in the samples. Liquid chromatography was performed on an AB ExionLC (AB SCIEX, Framingham, MA, USA) with an ACQUITY UPLC BEH Amide column (100 mm × 2.1 mm, 1.7 μm). Mass spectrometry was performed on an AB SCIEX Selex ION Triple Quad™ 6500 system, with an electrospray ionisation source, operating in both positive and negative ion modes. Nitrogen was employed as the collision gas. Targeted metabolites were analysed in multiple reaction monitoring (MRM) mode. The MRM pairs, delustering potentials, and collision energies were optimised for each analyte. Data acquisition and further analysis were conducted using Analyst software. SCIEX OS-MQ software was used to quantify all metabolites.

### Allograft tumour models

Male 615 mice (4–6 weeks old, purchased from SPF [Beijing] Biotechnology Co., Ltd., Beijing, China) were housed in specific-pathogen-free cages and used to construct allograft tumour models. Animal experiments were performed in accordance with the institution’s guidelines and animal research principles, and daily care was provided. After acclimatisation for 3 days in controlled environmental conditions, experiments were conducted. The single-blind method was adopted in our experiments. Animal experiments were conducted in compliance with animal use guidelines and were approved by the Laboratory Animal Ethics Committee of Ruijin Hospital (Approval No. SYXK2018-0023). Mouse forestomach carcinoma (MFC) cells (2 × 10^5^ suspended in 0.1 mL of PBS) were injected subcutaneously into 615 mice. Tumour growth was monitored every 3 days. From day 6, shSLC7A9 lentivirus (intratumourally), PT and 5-FU (intraperitoneally) were injected into the mice (n = 4). Tumour volume (mm^3^) was determined by measuring the longest diameter (a) and shortest width (b) and calculated using the following formula: volume (mm^3^) = 0.5 × a × b^2^. On day 18, the mice were euthanised, and the tumours were harvested. The average tumour weights of the dimethyl sulphoxide (DMSO)-treated group was denoted as a. Using the tumour weights of the PT + 5FU group (denoted as c1–c4) and the shSLC7A9 + PT + 5FU group (denoted as d1–d4), the inhibition rates were calculated as (a-c)/a∗100 and (a-d)/a∗100, respectively. Finally, a Student’s *t*-test was performed to compare the inhibition rates between the two groups.

### Establishment and culture of organoids from patients with gastric cancer

Patient-derived organoids (PDO) were established as previously described.^27^ Tumour tissues from patients with gastric cancer were removed and washed with PBS to remove adipose and connective tissues. The tissues were cut with a scalpel into 2 mm pieces and washed with PBS repeatedly. The remaining clear tissue was digested with PBS + ethylenediaminetetraacetic acid (2 mM) for 20 min at 4 °C with shaking. The tissue was collected, resuspended in 10 mL PBS, and repeatedly filtered through a 70 μm cell strainer (BD Biosciences, Franklin Lakes, NJ, USA). Following centrifugation, most of the supernatant was carefully aspirated and 100 μL of Matrigel was added to the pellet, being mindful to prevent the introduction of air bubbles. Approximately 500 to 1000 cells in 20 μL of Matrigel were plated in each well of a prewarmed 96-well plate. In every well, 0.1 mL of crypt culture medium was added, comprising advanced DMEM/F12 (Life Technologies) supplemented with serum-free B27 (1:50, Life Technologies), N2 (1:100, Life Technologies), N-acetylcysteine (50 mM, Sigma), recombinant murine epithelial growth factor (50 ng/mL; Peprotech, Rocky Hill, NJ, USA), Noggin (100 ng/mL, Peprotech), R-Spondin (1 μg/mL, Peprotech), Glutamax-I Supplement (1:100, Life Technologies), penicillin/streptomycin (400 μg/mL, Life Technologies), and HEPES (10 μM, Life Technologies). The cultures were maintained in a humidified incubator at 37 °C under 5% CO_2_. After incubation for 5 days, the organoids were transfected with shSLC7A9 or control lentivirus. The CellTiter-Lumi™ Plus II kit (C0057, Beyotime Biotechnology) was used to evaluate the viability of the organoids.

### Patient-derived xenograft model construction

The patient-derived xenograft (PDX) model was established using severe combined immunodeficient (SCID) mice as described previously.^28^ Fresh gastric cancer tissues were collected and washed thrice with PBS containing 1 × penicillin/streptomycin, followed by the removal of adipose tissue, connective tissue, and gastric mucus. Tumour tissues were then cut into 1–2 mm pieces using sterilised scissors and inoculated into SCID mice to establish the PDX model. Routine pathological analyses were performed on successfully established PDX tumours. Xenograft tumours were excised and repeatedly transplanted into new mice until they reached a sufficient volume for further experiments. Animal experiments were conducted in compliance with animal use guidelines and were approved by the Laboratory Animal Ethics Committee of Ruijin Hospital (Approval No. SYXK2018-0023).

### Statistical analysis

All statistical analyses were performed using GraphPad Prism (version 9.3.0; GraphPad, San Diego, CA, USA). Categorical data were evaluated using the chi-square test or Fisher’s exact test. Quantitative results are presented as the mean ± standard deviation. Differences between the two groups were analysed using the Student’s t-test. Comparisons among multiple groups were performed using one-way or two-way analysis of variance. Overall survival rates were estimated using the Kaplan–Meier method and compared using the log-rank test. Statistical significance was set at *p* < 0.05.

### Role of the funding source

The funding sources had no role in the study design; data collection, analyses, or interpretation; writing of the report; or the decision to submit the paper for publication.

## Results

### SLC7A9 expression was upregulated in gastric cancer tissues and indicated a poor prognosis

**Fig. 1:**
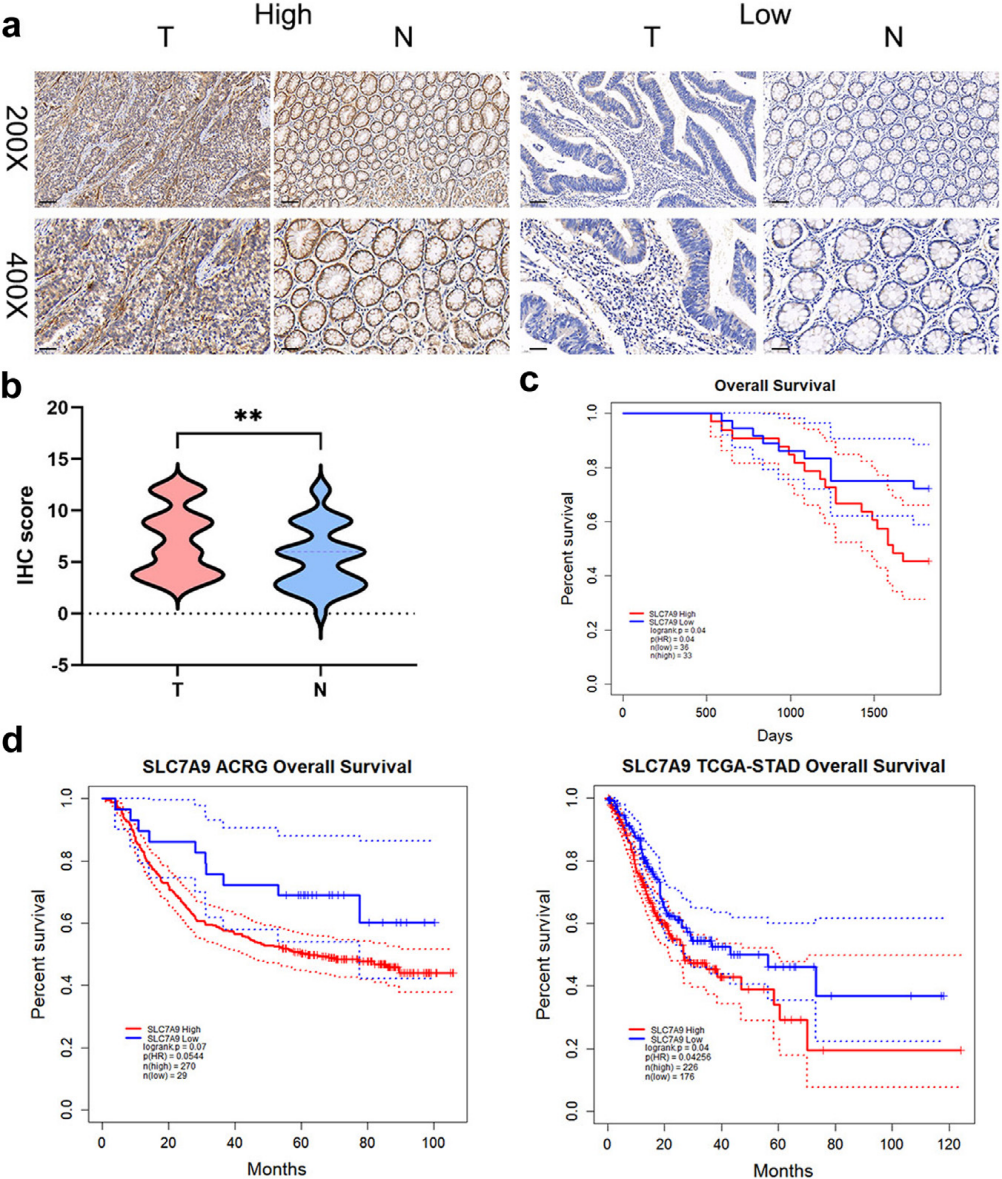
**SLC7A9 was upregulated in gastric cancer and related to poor prognosis. a**) and **b**) Representative images of IHC staining (**a**) and IHC score (**b**) of SLC7A9 in tumour tissues and non-tumour tissues of gastric cancer. 200x scale: 100 μm, 400x scale: 40 μm. Student’s t-tests. **c**) Survival outcomes of patients with gastric cancer of different SLC7A9 expression in Ruijin cohort. Log-rank (Mantel–Cox) test. **d**) Survival outcomes of patients with gastric cancer of different SLC7A9 expression in the ACRG and TCGA database. Log-rank (Mantel–Cox) test. ∗∗*p* < 0.01.

To identify the clinical significance of SLC7A9 in gastric cancer, the correlation between SLC7A9 expression level and clinicopathological parameters was analysed in 69 patients with gastric cancer (Table 1). The average immunohistochemistry (IHC) staining score for SLC7A9 expression was significantly higher in tumour tissues than in adjacent non-tumour tissues (Fig. 1a and b). A higher staining score for SLC7A9 in tumour tissues was correlated with rapid tumour recurrence (*p* = 0.0203, chi-square test) and an advanced TNM stage (*p* = 0.002, chi-square test), which indicated that SLC7A9 promoted resistance to chemotherapy and the progression of gastric cancer. No significant association was found between SLC7A9 expression levels and other parameters, such as age, sex, differentiation degree, and tumour invasion (Table 1). Patients with higher SLC7A9 expression levels in tumour tissues had worse 5-year overall survival outcomes than those with lower SLC7A9 expression levels (*p* = 0.04, log-rank [Mantel–Cox] test; Fig. 1c). Similar results were observed in the data collected from Asian Cancer Research Group (ACRG) and The Cancer Genome Atlas (TCGA) databases (ACRG, *p* = 0.0544; TCGA, *p* = 0.04256; Fig. 1d, log-rank [Mantel–Cox] test).

**Table 1:** The clinical characteristics of SLC7A9 high or low expressed patients.

Clinicopathologic parameters	Number of cases N = 69	SLC7A9 IHC		*p*-value
		Low (N = 36)	High (N = 33)	
**Age(years)**				0.4219
≤65	30	14	16	
>65	39	22	17	
**Sex**				0.7383
Male	51	26	25	
Female	18	10	8	
**Local invasion**				0.2334
T1, T2	17	11	6	
T3, T4	52	25	27	
**Lymph node metastasis**				0.0993
N0	19	14	5	
N1	12	7	5	
N2	19	7	12	
N3	19	8	11	
**Distant metastasis**				0.1401
No	61	34	27	
Yes	8	2	6	
**TNM stage**				0.002
I, II	30	22	8	
III, IV	39	14	25	
**Differentiation**				0.3322
Low	38	21	17	
Middle	25	13	12	
High	5	1	4	
**Recurrence or not**				
Recurrence after chemotherapy	24	7	17	0.0203
No recurrence after chemotherapy	41	25	16	

Note: One patient in the low group lacked information about tumour differentiation; four patients in low group did not receive chemotherapy after surgery.

### SLC7A9 expression was upregulated in *TP53*-deficient gastric cancer

**Fig. 2:**
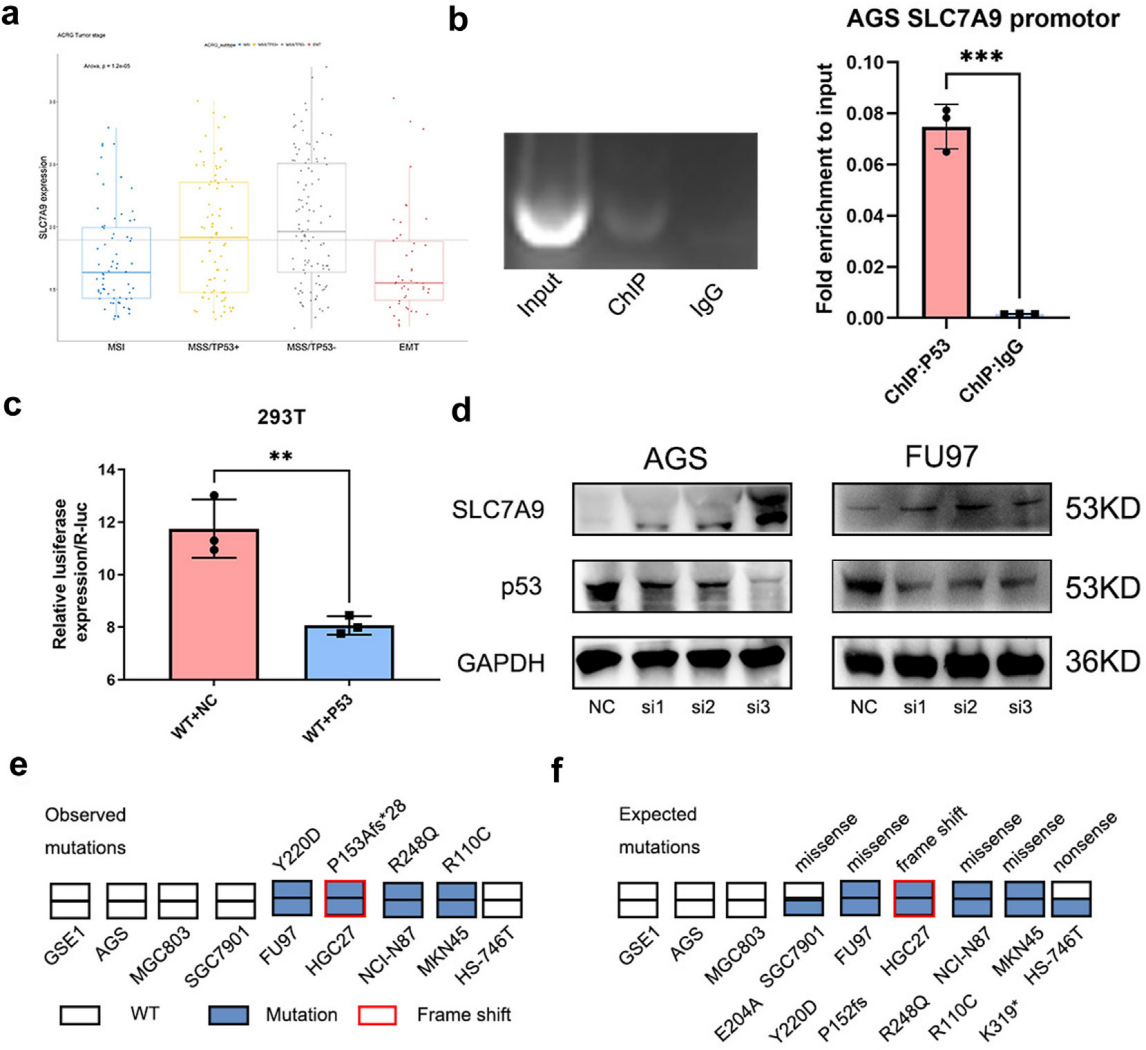
***TP53* transcriptionally repressed SLC7A9. a**) SLC7A9 expression in different ACRG types of gastric cancer. The bottom and top of the boxes were the 25th and 75th percentiles (interquartile range). The dotted line showed the average express of sample. One-way ANOVA. **b**) ChIP-PCR showing DNA fragments pulled down by p53 antibody in the SLC7A9 promoter. Student’s t-tests. **c**) The luciferase activity of the wild-type (WT) SLC7A9 promoter-driven luciferase reporter in the 293T cells with or without-*TP53* overexpression. Student’s t-tests. **d**) SLC7A9 and p53 expression in FU97 and AGS p53 knockdown cells. **e**) *TP53* status of 9 cell lines detected by Sanger sequencing. **f**) *TP53* status of 9 cell lines generated from database. ∗∗*p* < 0.01, ∗∗∗*p* < 0.001.

**Fig. 3:**
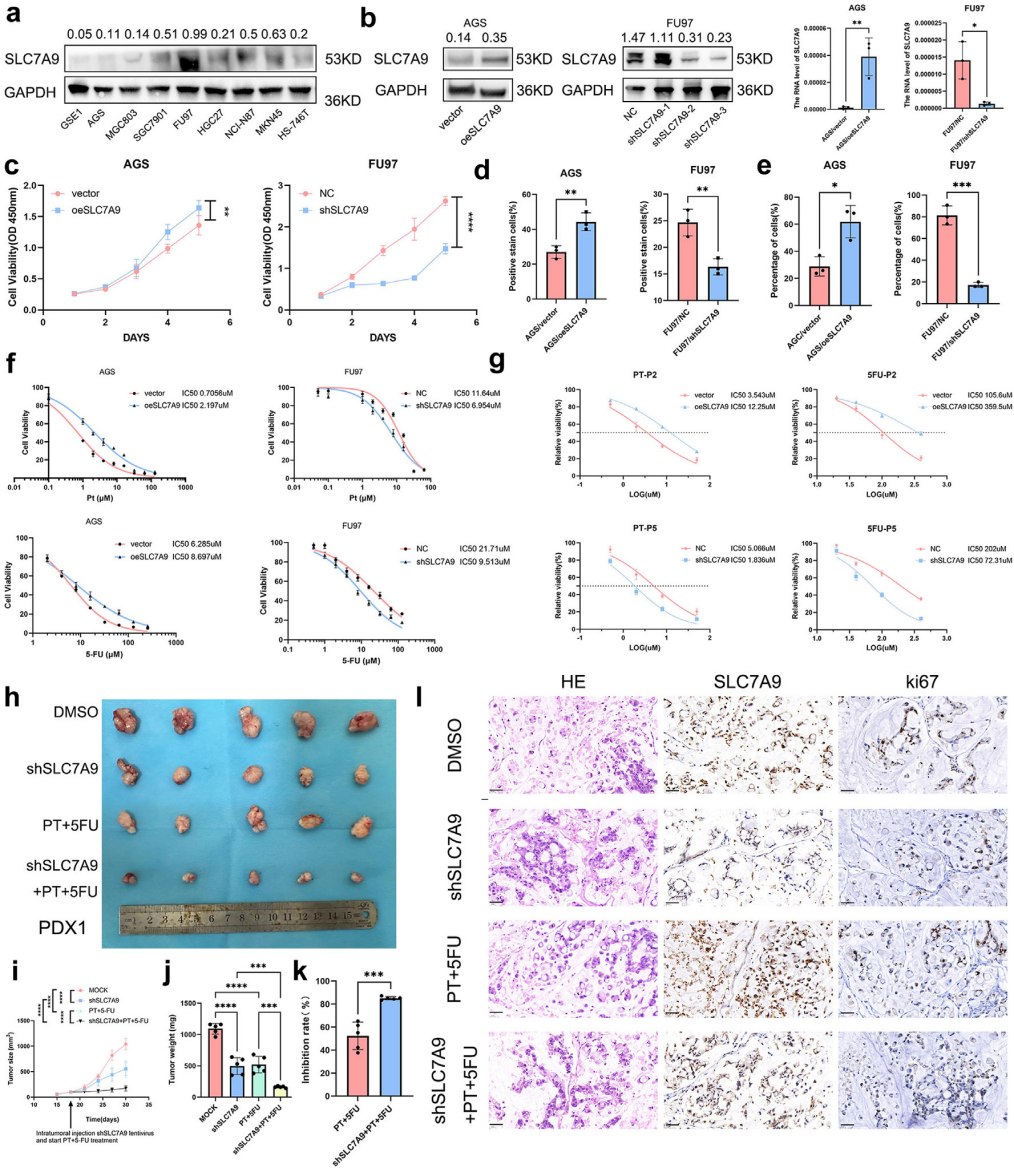
**SLC7A9 enhanced tumour cell proliferation and induced chemoresistance in gastric cancer cells. a**) SLC7A9 protein levels in eight gastric cancer cell lines and one immortalized stomach epithelial cell line (Western blot). **b**) SLC7A9 knockdown via shRNA in FU97 and overexpression in AGS. Student’s t-tests. **c**) CCK8 assay of cell proliferation. Two-way ANOVA. **d**) Relative quantification of EdU incorporation. Student’s t-tests. **e**) Relative quantification of Colony formation assay. Student’s t-tests. **f**) Inhibition curve of PT and 5-FU in indicated gastric cancer cells. **g**) Inhibition curve of PT and 5-FU in indicated gastric cancer organoids. **h**) Representative graphs of resected tumours from mice with subcutaneous tumours. PT: 6 mg/kg; 5-FU: 50 mg/kg. **i**) Subcutaneous tumour growth curves recorded using calipers at every 3 days after inoculation. Two-way ANOVA. **j**) Tumour weight recorded at the harvest time and plotted according to the treatment group. One-way ANOVA. **k**) Tumour inhibition rate of indicate groups. Student’s t-tests. **l**) Representative images of HE, SLC7A9 and ki67 IHC staining, scale bar 40 μm ∗*p* < 0.05, ∗∗*p* < 0.01, ∗∗∗*p* < 0.001, ∗∗∗∗*p* < 0.0001.

In our previous study, SLC7A9 was identified as the most significant DRG between gastric cancer tissues and normal control tissues using DRN analysis.^22^ Based on the ACRG data, we found that the transcript level of *SLC7A9* was higher in patients with microsatellite stable (MSS)/*TP53* (−) tumours than in patients with MSS/*TP53* (+) tumours, suggesting that the SLC7A9 expression level was related to *TP53* status (Fig. 2a). ChIP-PCR analysis showed that p53 bound directly to the *SLC7A9* promoter region, which was consistent with the JASPAR prediction (https://maayanlab.cloud/Harmonizome/gene_set/TP53/JASPAR+Predicted+Transcription+Factor+Targets) (Fig. 2b). A dual-luciferase reporter assay in HEK293T cells confirmed that p53 bound directly to the *SLC7A9* promoter and inhibited its transcriptional activity (Fig. 2c). SLC7A9 expression levels were significantly enhanced in AGS and FU97 gastric cancer cells upon the inhibition of p53 expression via small interfering (si) RNA (Fig. 2d). The correlation between SLC7A9 expression level and *TP53* status in the cell lines was further examined using Sanger sequencing of *TP53* exons 4–8 in nine cell lines (Fig. 2e). The results were consistent with those obtained from the data collected from online databases^29^ (https://tp53.cancer.gov; Fig. 2f). In *TP53*-wild-type cell lines, including GES1, AGS, MGC803, SGC7901, and HS-746T cells, SLC7A9 expression levels were downregulated. FU97, NCI–N87, and MKN45 cells had homozygous mutations in *TP53* and HGC27 cells had a homozygous frame shift mutation in *TP53*. The SLC7A9 expression levels were upregulated in these cells (Fig. 2, Fig. 3a). Collectively, these findings confirmed that *TP53* negatively regulated *SLC7A9* at the transcriptional level. Upon the loss of *TP53* function, SLC7A9 exhibited elevated expression levels.

### SLC7A9 promoted gastric cancer cell proliferation and induced chemoresistance

To explore the function of SLC7A9 in gastric cancer, the SLC7A9 expression levels were determined in eight gastric cancer cell lines (AGS, MKN45, HGC27, MGC803, NCI–N87, FU97, SGC7901, and HS-746T cells) and one immortalised stomach epithelial cell line (GES1 cells; Fig. 3a). We knocked down SLC7A9 expression with lentivirus-mediated shRNA interference in FU97 cells, which had the highest expression level of SLC7A9, and found that cell proliferation was significantly inhibited as a result. Upon transfection of an SLC7A9-overexpression vector (oeSLC7A9) into AGS cells, which had the lowest expression level of SLC7A9, cell proliferation was enhanced (Fig. 3b–e and Supplementary Fig. S1a and b).

GSEA was conducted between the high- and low-SLC7A9-expression groups (203 vs. 204) in the TCGA-STAD cohort (Supplementary Fig. S1c–e). Pathways related to drug metabolism and resistance were found to be enriched (Supplementary Fig. S1c–d). ABC transporters are important for mediating cellular drug influx, and the cytochrome P450 system includes a series of essential enzymes involved in drug transition and elimination. Our results showed that high SLC7A9 expression level was positively correlated with the cytochrome P450 (NES = 1.9003, *p* = 0.006, Fisher’s exact test) and the ABC transporter (NES = 1.6799, *p* = 0.025, Fisher’s exact test) pathways (Supplementary Fig. S1e). For patients with gastric cancer, PT, and 5-FU are used as the first-line drugs.^2^ Consistent with the GSEA results, SLC7A9 knockdown significantly enhanced the sensitivity of FU97 cells to PT and 5-FU, whereas SLC7A9 overexpression mediated the resistance of AGS cells to PT and 5-FU (Fig. 3f).

To further confirm the role of SLC7A9 in mediating drug resistance in human gastric cancer, we established PDO models using gastric cancer tissues. Immunofluorescence staining showed that the SLC7A9 expression levels varied in the seven PDOs (Supplementary Fig. S2a). Among the seven PDOs, we transfected virus into organoids from patient 2 (P2, SLC7A9^low^) and patient 5 (P5, SLC7A9^high^) and established P5/shSLC7A9, P5/shNC, P2/oeSLC7A9, and P2/vector organoids. These organoids were then treated with PT or 5-FU and their viability was determined. PT and 5-FU exerted a more potent effect on P5/shSLC7A9 organoids than on P5/shNC organoids, whereas the antitumour effects of PT and 5-FU were attenuated in P2/oeSLC7A9 organoids (Fig. 3g and Supplementary Fig. S2b and c).

Further, we examined the *in vivo* effects of shSLC7A9 on the efficacy of chemotherapy drugs. First, we determined the expression level of SLC7A9 in three PDX cases and found that SLC7A9 was expressed at the highest level in PDX1 (Supplementary Fig. S2d). Therefore, we established the PDX1 gastric cancer model and injected control and shSLC7A9 lentiviruses at multiple sites around the PDX tumours. When the tumour reached 100 mm^3^, the mice were administered PT (6 mg/kg) + 5-FU (50 mg/kg) intraperitoneally every 2 days. After 30 days, the mice were euthanised and the tumours were collected (Fig. 3h). We found that both SLC7A9 knockdown and chemotherapy inhibited tumour growth, and the dual treatment showed the maximum therapeutic effect (Fig. 3h–k). IHC analysis showed a decreased number of ki67-positive cells in shSLC7A9-and chemotherapy-treated tumours compared to control tumours, and cells positively stained for ki67 were fewest in shSLC7A9 + chemotherapy–treated tumours (Fig. 3l).

To represent the whole microenvironment of gastric cancer, the *in vivo* effects of shSLC7A9 on the efficacy of chemotherapy drugs were also evaluated in immunocompetent mice treated with MFC cells. MFC cells were subcutaneously inoculated into the flanks of 615 mice (Supplementary Fig. S3a). When the tumour reached 100 mm^3^, control or shSLC7A9 lentiviruses were injected at multiple sites around the tumours. The mice were administered PT (6 mg/kg) + 5-FU (50 mg/kg) intraperitoneally every 2 days. After 18 days, the mice were euthanised and the tumours were collected (Supplementary Fig. S3a). We found that both SLC7A9 knockdown and chemotherapy inhibited tumour growth, and the dual treatment showed the maximum therapeutic effect (Supplementary Fig. S3b–d). IHC analysis showed a decreased number of ki67-positive cells in shSLC7A9-and chemotherapy-treated tumours compared to the number in control tumours, and cells positively stained for ki67 were fewest in shSLC7A9 + chemotherapy drug–treated tumours (Supplementary Fig. S3e). These findings indicated that SLC7A9 played an oncogenic role in gastric cancer and made mice to chemoresistance when they had normal immunity.

### SLC7A9 inhibited ferroptosis and maintained GSH metabolism via cystine transport

**Fig. 4:**
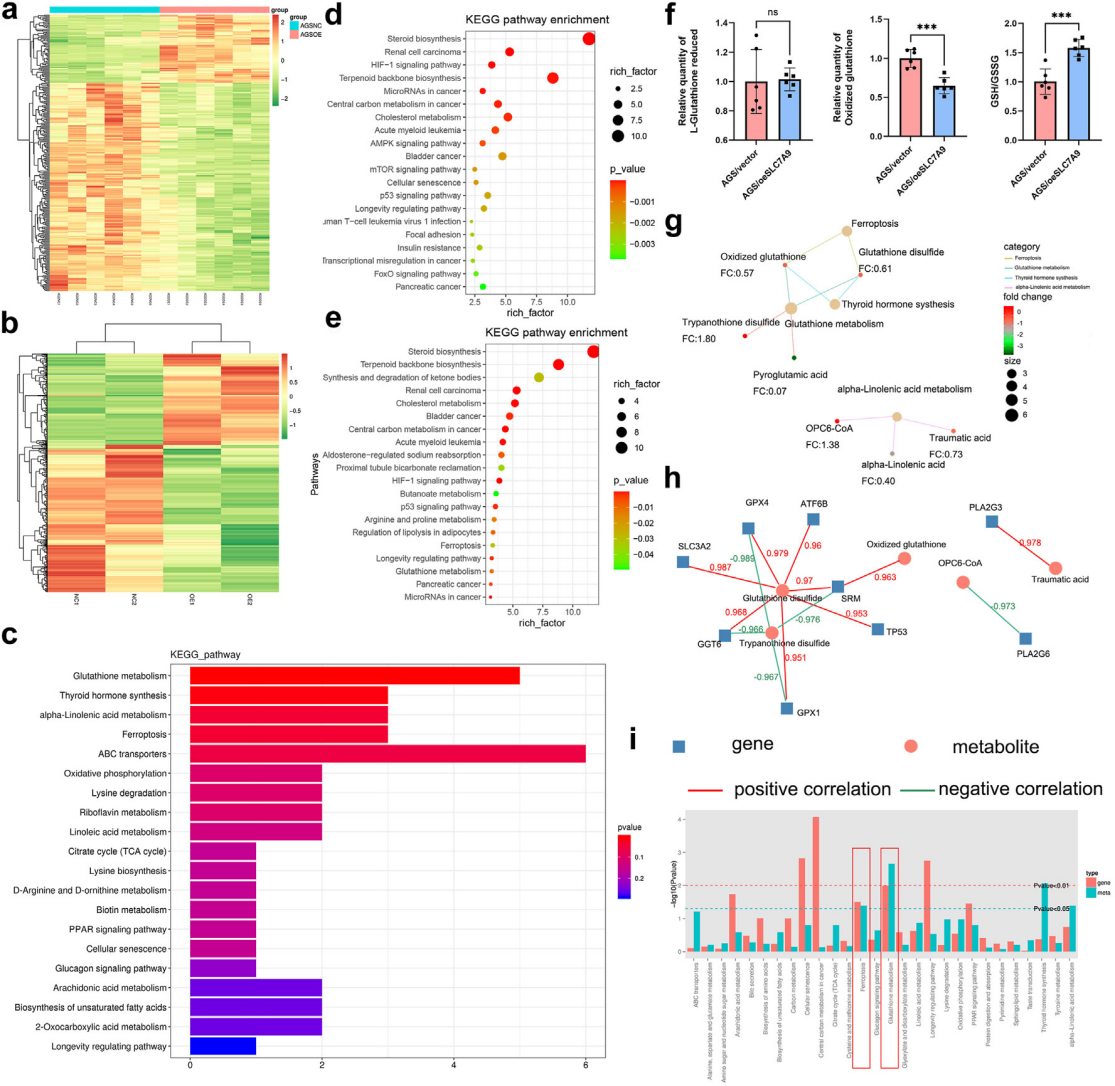
**SLC7A9 inhibited ferroptosis and maintained glutathione metabolism in gastric cancer cells. a**) Heatmap of total differential metabolites from metabolism-seq analyses. **b**) Heatmap of total differential genes from RNA-seq analyses. **c**) KEGG pathway enrichment based on DEM. **d**) KEGG pathway enrichment based on DEGs was ranked in descending order by *p*-value order. **e**) KEGG pathway enrichment based on DEGs was ranked in descending order by enrichment score. **f**) GSH, GSSG and GSH/GSSG ratio of metabolome data between AGS/oeSLC7A9 and AGS/vector group. Student’s t-tests. **g**) A diagram of the relationship between KEGG pathway and relative metabolites. **h**) A diagram of the relationship between metabolites and genes. **i**) The top 20 up-regulated and down-regulated pathways based on a combination of DEM and DEG. ns, not significant; ∗∗∗, *p* < 0.001.

There is evidence supporting the potential link between the SLC7A9 expression level and cellular metabolism.^18^, ^19^, ^20^, ^21^ Hence, we conducted RNA sequencing (GSE212900) and untargeted metabolome profiling of both AGS/oeSLC7A9 and AGS/vector cells (Fig. 4a and b). Differentially expressed genes (DEGs, fold change [FC] > 1.5, FDR < 0.01) and metabolites (DEMs, FC > 1, *p* < 0.01, and VIP >1, Fisher’s exact test) were identified. The top 20 biological pathways enriched in the Kyoto Encyclopedia of Genes and Genomes (KEGG) database based on the DEGs and DEMs were shown in Fig. 4c–e. SLC7A9 is known as a cystine transporter. According to the metabolome and transcriptome data, SLC7A9 is mainly involved in GSH metabolism and ferroptosis in gastric cancer cell lines. We further analysed the GSH and GSSG levels in the metabolome data. The results showed that there was no significant difference in GSH levels between the two groups of cells, while the GSSG level was significantly lower in the AGS/oeSLC7A9 group than in the AGS/NC group, suggesting that the reduction status of AGS/oeSLC7A9 group was improved (Fig. 4f). The node diagram of the DEG-enriched pathways indicated a related network between the AGS/oeSLC7A9 and AGS/vector groups (Fig. 4g). The node diagram constructed using DEMs and their related genes indicated that SLC7A9 levels were correlated with GPX4 levels and p53 levels were correlated with GSSG levels (Fig. 4h). Integrative analysis of transcriptomic and metabolomic data indicated that ferroptosis- and GSH-metabolism-associated genes and metabolites were differentially expressed between the SLC7A9-overexpression and control groups (GSH metabolism, *p* = 0.010 for DEGs, *p* = 0.002 for DEMs; ferroptosis, *p* = 0.031 for DEGs, *p* = 0.041 for DEMs; Fig. 4i; Fisher’s exact test).

**Fig. 5:**
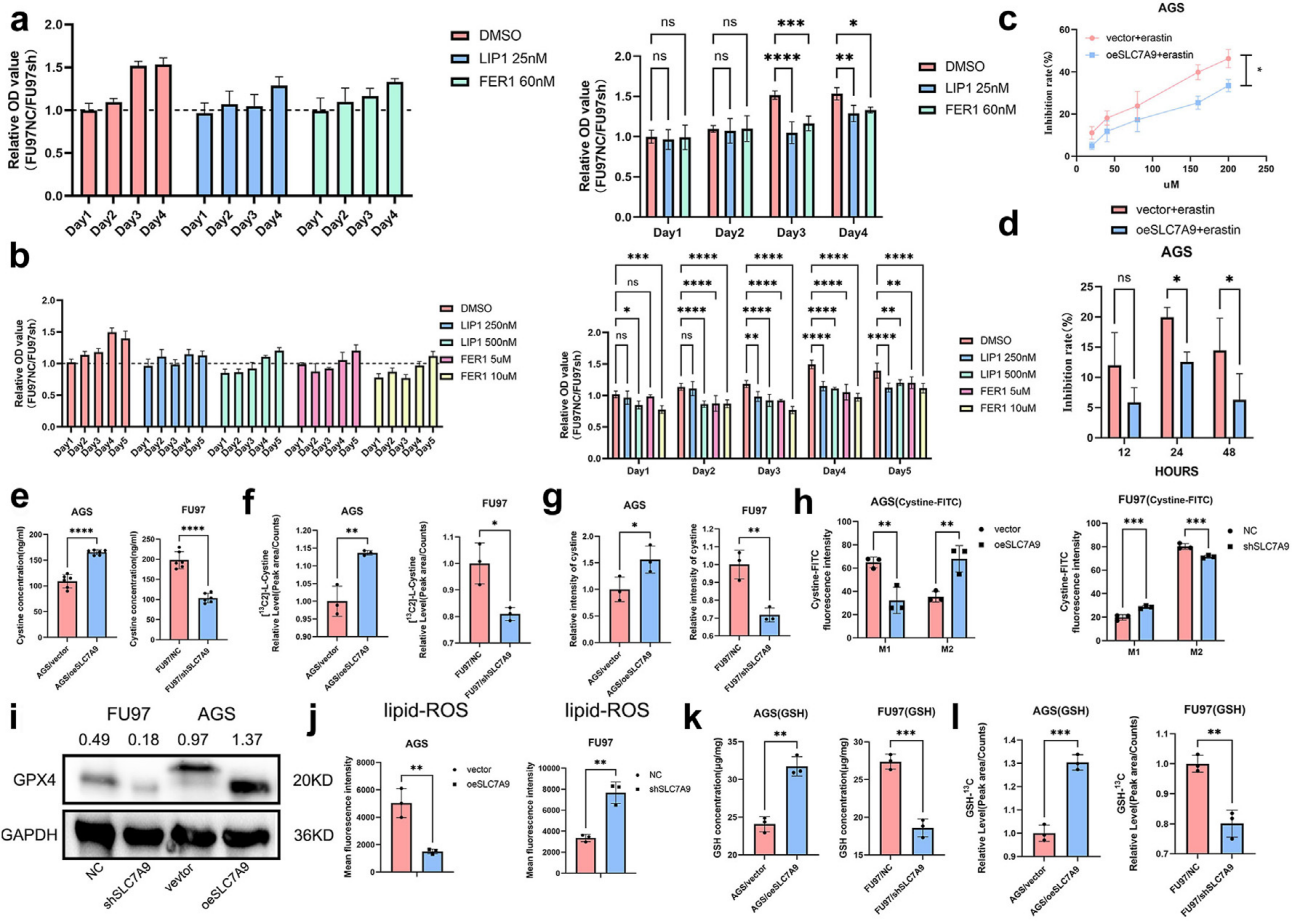
**SLC7A9 inhibited ferroptosis in gastric cancer cells. a**) Cell proliferation ratio (FU97/NC and FU97/shSLC7A9) (ferroptosis inhibitor was added on day 0, day 2 and day 4). Two-way ANOVA. **b**) Cell proliferation ratio (FU97/NC and FU97/shSLC7A9) (ferroptosis inhibitor was added only on day 0 without changing the culture medium). Two-way ANOVA. **c**) Inhibition rate of gastric cancer cells with erastin administration for 24h. Two-way ANOVA. **d**) Inhibition rates of gastric cancer cells at 12, 24 and 48 h after erastin (40 μM) administration. Two-way ANOVA. **e**) Cystine concentration (ng/ml) in each group. Student’s t-tests. **f**) Peak area of ^13^C-cystine in each group. Student’s t-tests. **g**) Relative quantification of cystine-FITC fluorescence. Student’s t-test. **h**) Relative quantification of cell cytometry of cystine-FITC positive cells. M1 represents cells without uptake of cystine-FITC and M2 represents cells with uptake of cystine-FITC. Two-way ANOVA. **i**) The levels of GPX4 in indicated cell lines were determined by Western blotting. **j**) Relative quantification of lipid-ROS mean fluorescence intensity in each group. ROS probe: C11-BODIPY. Student’s t-tests. **k**) GSH concentration (μg/mg) in each group. Detection: GSH assay kit. Student’s t-tests. **l**) Peak area of ^13^C-GSH in each group. Student’s t-tests. ns, not significant, ∗*p* < 0.05, ∗∗*p* < 0.01, ∗∗∗*p* < 0.001, ∗∗∗∗*p* < 0.0001.

Ferroptosis exerts important effects on cancer cell clearance.^5^ To illustrate the effect of SLC7A9 on ferroptosis, gastric cancer cells were treated with ferroptosis inhibitors (liproxstatin-1 and ferrostatin-1) or a ferroptosis inducer (erastin), and cell viability was observed. SLC7A9 knockdown promoted ferroptosis and inhibited FU97 cell proliferation. The FU97-NC/FU97-shSLC7A9 OD_450_ ratio was approximately 1.5 on day 3 (Fig. 5a). The ferroptosis inhibitor treatment slightly promoted the growth of FU97-shSLC7A9 cells, and the growth disparity was less pronounced between FU97-NC and FU97-shSLC7A9 cells treated with ferroptosis inhibitors (relative OD_450_ values were approximately 1–1.2 on day 3) than between FU97-NC and FU97-shSLC7A9 cells treated with DMSO (Fig. 5a), indicating that ferroptosis inhibitors rescued SLC7A9-knockdown-mediated ferroptosis. Similar results were observed at higher concentrations of the ferroptosis inhibitors (Fig. 5b). These results suggested that blocking ferroptosis reversed the inhibitory effects of SLC7A9 knockdown on cell growth. Moreover, SLC7A9 overexpression attenuated the cytotoxic effect of erastin-induced ferroptosis of gastric cancer cells (Fig. 5c and d). Taken together, these results indicated that SLC7A9 inhibited ferroptosis, whereas SLC7A9 knockdown promoted ferroptosis.

Mechanistically, ferroptosis reflects lipid peroxide accumulation and an imbalanced redox equilibrium in cancer cells.^5^^,^^6^ Cystine is an essential substrate for GSH production, and GSH metabolism plays a major role in the regulation of the redox equilibrium.^30^ Therefore, we hypothesised that SLC7A9 regulated GSH metabolism and the redox equilibrium by functioning as a cystine transporter. SLC7A9 knockdown in FU97 cells resulted in a lower cystine concentration, a smaller proportion of cystine-FITC-positive cells, and lower intracellular fluorescence intensity of cystine-FITC compared to those values in the control group (Fig. 5e–h and Supplementary Fig. S4a–d). In contrast, SLC7A9 overexpression in AGS cells resulted in higher cystine concentrations, a larger proportion of cystine-FITC-positive cells, and a higher intracellular fluorescence intensity of cystine-FITC compared to those values in the control group (Fig. 5e–h and Supplementary Fig. S4a–d). Metabolic flow experiments, by adding ^13^C-labelled metabolites to the cell culture medium, were performed to directly display the cellular flow of cystine and GSH. SLC7A9 knockdown in FU97 cells resulted in a lower peak area of ^13^C-cystine compared to its area in control cells (Fig. 5f). In contrast, overexpression of SLC7A9 in AGS cells resulted in a higher peak area of ^13^C-cystine compared to its area in control cells (Fig. 5f). GPX4, an enzyme that converts toxic lipid hydroperoxides into non-toxic lipid alcohols, prevents ferroptotic cell death,^12^ and its activity is regulated by the cystine/glutamate antiporter system X_C_^−^.^11^^,^^12^^,^^14^ We also found that SLC7A9 knockdown inhibited GPX4 expression, whereas SLC7A9 overexpression elevated GPX4 expression level (Fig. 5i). SLC7A9 knockdown increased lipid peroxide levels in FU97 cells, whereas SLC7A9 overexpression reduced lipid peroxide levels in AGS cells (Fig. 5j and Supplementary Fig. S4c). In addition, SLC7A9 overexpression increased the GSH concentration and GSH/GSSG ratio compared to the ratios in the control group, whereas SLC7A9 downregulation decreased both the GSH concentration and GSH/GSSG ratio (Fig. 5k and Supplementary Fig. S4d and e). SLC7A9 knockdown in FU97 cells resulted in a lower peak area of ^13^C-GSH compared to its area in control cells, indicating less GSH was synthesised with lower SLC7A9 expression levels, while SLC7A9 overexpression in AGS cells resulted in a higher peak area of ^13^C-GSH compared to its area in control cells (Fig. 5l). Overall, these findings suggested that SLC7A9 inhibited ferroptosis by transporting cystine into cells, regulating GPX4 expression, and modulating GSH levels, thereby maintaining the cellular redox status.

### SLC7A9 exerted its effect on cystine influx and GSH production independently of SLC7A11

**Fig. 6:**
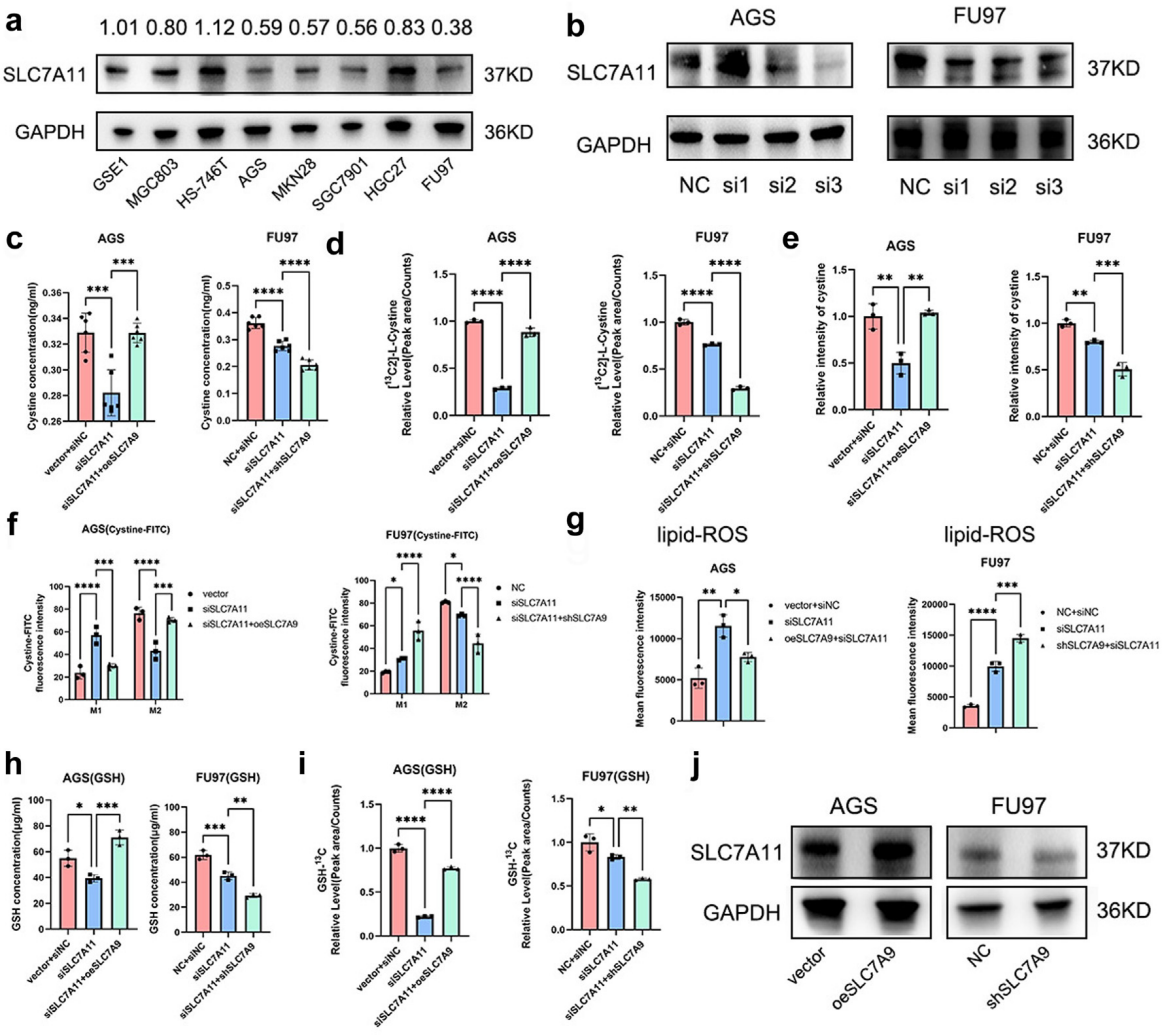
**SLC7A9 affected cystine influx and GSH production independently of SLC7A11. a**) SLC7A11 protein levels in indicated cell lines (Western blot). **b**) SLC7A11 knockdown via siRNA in FU97 and AGS. **c**) Cystine concentration (ng/ml) in each group. One-way ANOVA. **d**) Peak area of ^13^C-cystine in each group. One-way ANOVA. **e**) Relative quantification of cystine-FITC fluorescence. One-way ANOVA. **f**) Relative quantification of cell cytometry of cystine-FITC positive cells. M1 represents cells without uptake of cystine-FITC and M2 represents cells with uptake of cystine-FITC. Two-way ANOVA. **g**) Relative quantification of mean fluorescence intensity in each group. ROS probe: C11-BODIPY. One-way ANOVA. **h**) GSH concentration (μg/ml) in each group. One-way ANOVA. **i**) Peak area of ^13^C-GSH in each group. One-way ANOVA. **j**) The levels of SLC7A11 in SLC7A9 overexpression or knockdown cell lines (Western blotting). ∗*p* < 0.05, ∗∗*p* < 0.01, ∗∗∗*p* < 0.001, ∗∗∗∗*p* < 0.0001.

SLC7A11 is a key factor in the regulation of ferroptosis and cancer development.^17^ Therefore, we explored whether SLC7A11 was involved in the role of SLC7A9 in inhibiting ferroptosis. *SLC7A11* transcripts were detected in seven gastric cancer cell lines and one immortalised stomach epithelial cell line. SLC7A11 was knocked down in AGS and FU97 cells using siRNA (Fig. 6a and b). The cystine concentration and cystine-FITC fluorescence intensity decreased with SLC7A11 knockdown, whereas SLC7A9 overexpression restored cystine influx in AGS cells (Fig. 6c–f and Supplementary Fig. S4f–g). In FU97/siSLC7A11 cells, cystine influx was inhibited, and this was further aggravated by SLC7A9 knockdown. SLC7A11 knockdown in AGS cells also resulted in a lower peak area of ^13^C-cystine, which was recovered by SLC7A9 overexpression (Fig. 6d). These findings indicated that the effect of SLC7A9 on cystine transport was independent of SLC7A11 (Fig. 6c–f and Supplementary Fig. S4f–g). It is well documented that SLC7A11 inhibition increases lipid peroxide levels. As expected, SLC7A9 overexpression reversed this inhibition by SLC7A11, whereas SLC7A9 knockdown enhanced this inhibitory effect (Fig. 6g and Supplementary Fig. S4h). SLC7A11 inhibition decreased GSH levels and GSH/GSSG ratio in FU97 and AGS cells, and this was reversed by SLC7A9 overexpression, whereas SLC7A9 knockdown further decreased GSH levels and GSH/GSSG ratio (Fig. 6h and Supplementary Fig. S4i and j). SLC7A11 knockdown in FU97 cells resulted in a lower peak area of ^13^C-GSH, and this was further aggravated by SLC7A9 knockdown. SLC7A11 knockdown in AGS cells also resulted in a lower peak area of ^13^C-GSH, which was recovered by SLC7A9 overexpression (Fig. 6i). SLC7A11 expression was not affected with SLC7A9 knockdown in FU97 or overexpression in AGS. These results showed that SLC7A9 did not interfere with SLC7A11 expression (Fig. 6j). Collectively, these findings indicated that SLC7A9 played a role in cystine transport and GSH production independently of SLC7A11.

### SLC7A9 suppressed ferroptosis and resisted the effect of erastin in PDO and PDX models

**Fig. 7:**
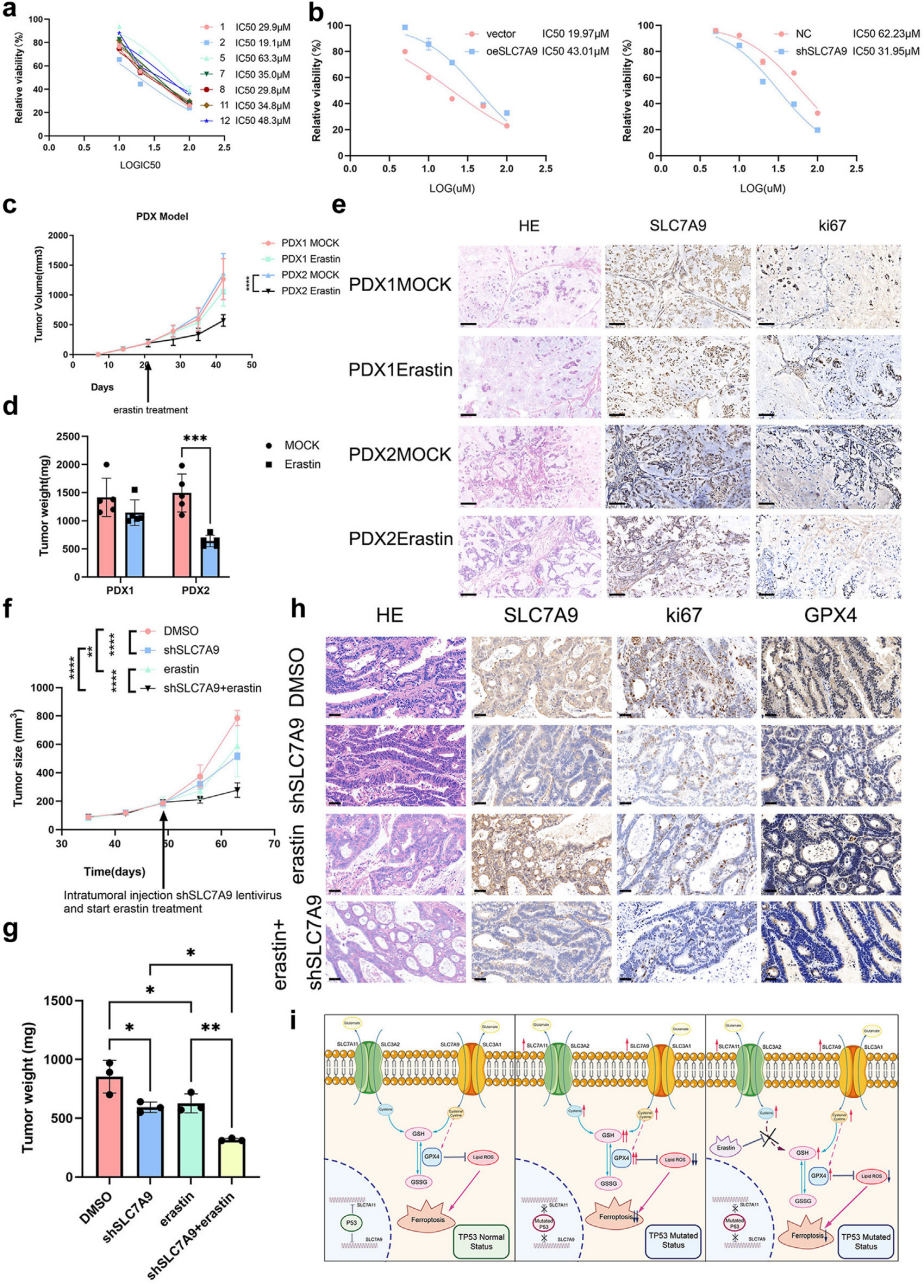
**SLC7A9 reversed tumour killing effect of erastin in PDO and PDX models and shSLC7A9 sensitized erastin *in vivo*. a**) IC_50_ of 7 PDOs treated with erastin. **b**) IC_50_ of erastin treated PDO with SLC7A9 knockdown or overexpression. **c**) Subcutaneous tumour dimensions recorded using calipers at every 7 days after inoculation. Two-way ANOVA. **d**) Tumour weight recorded at the harvest time and plotted according to the treatment group. Two-way ANOVA. **e**) Representative images of HE, SLC7A9 and ki67 IHC staining, scale bar 100 μm. erastin: 30 mg/kg. **f**) Subcutaneous tumour dimensions were recorded using calipers at every 7 days. Two-way ANOVA. **g**) Tumour weight was recorded at time of harvest and plotted according to treatment group. One-way ANOVA. **h**) HE, SLC7A9, ki67 and GPX4 images of each group, scale bar 40 μm. **i**) Working models of p53 regulation on SLC7A9 function and ferroptosis. Left: p53 regulates SLC7A9 and SLC7A11 in *TP53* wild-type cells. Mid: p53 regulates SLC7A9 and SLC7A11 in *TP53* mutated cells. Right: SLC7A9 regulates ferroptosis independently of SLC7A11. ∗*p* < 0.05, ∗∗*p* < 0.01, ∗∗∗*p* < 0.001, ∗∗∗∗*p* < 0.0001.

As previously mentioned, PDO models were established using gastric cancer tissues. To further confirm the oncogenic role of SLC7A9 in human gastric cancer, we treated cells with erastin and evaluated the growth of the organoids. The growth rates of the PDO implied that erastin inhibited the viability of P2 organoids (low SLC7A9 expression level) more efficiently than that of P5 organoids (high SLC7A9 expression level), and PDO with higher SLC7A9 expression levels displayed higher IC_50_ values for erastin (Fig. 7a and Supplementary Fig. S5a). Moreover, erastin inhibited the viability of P5/shSLC7A9 organoids more efficiently than that of P5/shNC organoids, whereas the antitumour effect of erastin was attenuated in P2/oeSLC7A9 organoids (Fig. 7b and Supplementary Fig. S5b and c).

Based on the *in vitro* findings described above, the *in vivo* effects of SLC7A9 overexpression were evaluated. We established PDX gastric cancer models and intraperitoneally injected erastin on day 21 (30 mg/kg administered every 2 days; Fig. 7c and Supplementary Fig. S6a). After 42 days, the mice were euthanised, tumours were collected, and SLC7A9 expression was evaluated by IHC (Fig. 7d–e). The growth of tumours with high expression levels of SLC7A9 was less affected by erastin treatment than the growth of tumours with low SLC7A9 expression levels (*p* = 0.0013; Fig. 7d and Supplementary Fig. S6a and b, Student’s t-test). IHC analysis showed an increased number of ki67-positive cells in SLC7A9^high^ tumours than in SLC7A9^low^ tumours treated with erastin (Fig. 7e).

To examine the therapeutic potential of SLC7A9, control and shSLC7A9 lentiviruses were injected at multiple sites around the PDX tumours, and erastin was injected intraperitoneally on day 49 (30 mg/kg administered every 2 days; Fig. 7f and Supplementary Fig. S6c). After 63 days, the mice were euthanised, the tumours were collected, and SLC7A9 expression was evaluated by IHC (Fig. 7f and g). We found that either SLC7A9 knockdown or erastin treatment inhibited tumour growth, and dual treatment showed the maximum therapeutic effect (Fig. 7f–g and Supplementary Fig. S6c and d). IHC analysis showed a decreased number of ki67-positive cells in shSLC7A9-and erastin-treated tumours than in control tumours, and ki67-positive cells were fewest in shSLC7A9 + erastin–treated tumours (Fig. 7g). GPX4-positive cells exhibited the same tendency as ki67-positive cells (Fig. 7g). In conclusion, these findings confirmed that SLC7A9 drove ferroptosis resistance and that SLC7A9 knockdown sensitised gastric cancer cells to ferroptosis *in vivo*.

## Discussion

Ferroptosis, as a form of RCD, is a powerful tumour-suppression mechanism.^5^^,^^31^ Ferroptosis is correlated with cellular iron metabolism and has been recently discovered as a new iron-dependent form of RCD induced by natural stimuli and synthetic agents.^6^^,^^32^ Redox-active iron and PUFA oxidation promote lipid peroxide production and accumulation,^33^ and GSH-dependent GPX4 expression may be an important checkpoint in ferroptosis.^13^ GPX4 appears to be the dominant antioxidant enzyme that utilises intracellular GSH to prevent toxic lipid peroxide accumulation.^34^^,^^35^ GSH production depends on the transport of cystine into the cytoplasm via the transmembrane cystine/glutamate antiporter system X_C_-^36^ and cysteine/cystine transporters (SLC1A4, SLC1A5, and SLC3A1).^13^^,^^15^^,^^37^, ^38^, ^39^ SLC3A1, an auxiliary subunit of the rBAT–b (0,+)AT1 (SLC7A9) transporter system, is linked to the capacity of breast cancer cells to maintain their redox state by promoting the accumulation of reduced GSH, thereby decreasing lipid peroxide levels and maintaining cell survival.^40^^,^^41^ In this study, we found that the SLC7A9 expression level correlated with both GSH metabolism and ferroptosis in gastric cancer cells. A higher expression level of SLC7A9 resulted in more cystine transported into the cytoplasm, increased GSH production, and promoted GPX4 protein synthesis, consequently suppressing lipid peroxide accumulation and ferroptosis. Cellular cystine intake has been shown to promote GPX4 protein synthesis via the Rag-mTORC1-4EBP pathway,^42^ which is consistent with our observations.

p53 regulates ferroptosis in a variety of ways. p53 has been reported to inhibit cystine uptake and promote ferroptosis by transcriptional or non-transcriptional repression of SLC7A11.^15^^,^^43^ p53 can also activate the expression of SAT1, thereby inducing lipid peroxidation and ferroptosis upon oxidative stress.^44^ Moreover, p53 promotes ferroptosis through modulation of GLS2, PTGS2, FDXR, DPP4, and noncoding RNAs.^45^ We found that SLC7A9 expression levels were elevated in *TP53*-mutated gastric cancer, and further, that p53 can transcriptionally inhibit *SLC7A9*. Since both SLC7A11 and SLC7A9 are present in gastric cancer cells, it is necessary to reveal the relationship between these two proteins. We demonstrated that SLC7A9 inhibited ferroptosis via an SLC7A11-independent mechanism. Moreover, the SLC7A9 expression level did not affect the SLC7A11 expression level. In our previous study, DRN analysis showed that SLC7A9, but not SLC7A11, was a key regulator in gastric cancer.^22^ Therefore, we speculated that SLC7A9 played a more important role in gastric cancer than SLC7A11.

Chemotherapy has been a classical cancer treatment for decades. Although millions of patients have benefited from chemotherapy, chemoresistance and subsequent tumour relapse have emerged as significant challenges.^46^ As an autonomous protective mechanism, ferroptosis eliminates precancerous cells exposed to metabolic stress or nutrient deprivation.^31^ It is believed that ferroptosis plays an increasingly important role in anticancer therapy. Ferroptosis not only helps cancer cells overcome resistance to chemotherapy, but it also reverses resistance to radiotherapy, immunotherapy, and targeted therapy.^47^, ^48^, ^49^ Recent studies have found that the system X_C_-/GPX4 axis participates in tumour chemoresistance.^50^ System X_C_- inhibition induces ferroptosis and restores cell sensitivity to chemotherapy in pancreatic ductal adenocarcinoma.^50^ Previous studies have shown that chemotherapy-resistant tumour cells are sensitive to ferroptosis inducers, which also provides the possibility for the clinical development of ferroptotic drugs.^47^ Furthermore, a number of clinical or preclinical investigations aimed at inducing ferroptosis are included in the ClinicalTrials database (https://clinicaltrials.gov/). For example, altretamine, cisplatin, and lapatinib are undergoing or have completed clinical trials as ferroptosis inducers targeting GPX4/GSH or iron metabolism for the management of HIV-related cancer, urothelial cancer, and metastatic breast cancer (study IDs NCT00002936, NCT04574960, and NCT00667251, respectively).^51^ Our results showed that SLC7A9 expression mediated cell resistance to chemotherapy. SLC7A9 knockdown sensitised gastric cancer cells to PT and 5-FU in PDO, Allograft tumour models and PDX models, which recapitulate gastric cancer characteristics in the laboratory. As SLC7A11 and SLC7A9 are located on the cellular membrane and play vital roles in ferroptosis, they offer an opportunity to develop targeted drugs. The combination of ferroptosis-targeting drugs and classical treatment modalities (chemotherapy, radiotherapy, and immunotherapy) holds great potential as a therapeutic strategy.

There are some limitations of our study. The clinical applications of inhibiting SLC7A9 expression to promote chemotherapy sensitivity are not described in this manuscript. In view of this, we propose two future research directions. First, siRNA drugs have been approved for clinical use by the Food and Drug Administration (FDA).^52^ Therefore, the design and chemical modification of siRNAs and siRNA-delivery systems may address this limitation. Second, we plan to explore functional inhibitory compounds targeting SLC7A9 or both SLC7A9 and SLC7A11 dual-targeted functional inhibition compounds through computer-aided drug design using compounds in the FDA library to achieve therapeutic translation of our research findings. Although the expression level of SLC7A9 is higher in gastric cancer than in normal tissue, there is a lack of appropriate methods to downregulating its expression. Therefore, we plan to explore the subtypes of gastric cancer with high expression levels of SLC7A9 through multi-omics analysis. In addition, we plan to design anti-SLC7A9 and anti-SLC7A11 antibody-drug conjugates in a follow-up study, to improve drug targeting of gastric cancer.

In conclusion, SLC7A9 promotes gastric cancer progression by inhibiting ferroptosis via the regulation of cystine and glutamate transport, GSH metabolism, and the redox equilibrium. Our findings indicate that SLC7A9 acts as a ferroptosis inhibitor independent of SLC7A11 (Fig. 7i). Therefore, therapeutic interventions targeting SLC7A9 are promising for sensitising gastric cancer cells to ferroptosis, and ultimately improving the prognosis of patients with gastric cancer.

## Contributors

Conceptualisation: Bingya Liu, Yuan–Yuan Li, Wentao Dai. Data duration: Haoran Feng, Junxian Yu. Formal analysis: Xiongyan Wu, Junyi Hou. Methodology: Zhuoqing Xu, Jianfang Li, Liping Su. Investigation: Haoran Feng, Junxian Yu, Qingqing Sang. Resources: Changyu He, Jiazeng Xia, Chao Yan, Zhenggang Zhu. Software: Qingqing Sang, Nan Zhu, Beiqin Yu, Yunqin Chen. Supervision: Bingya Liu, Yuan–Yuan Li, Wentao Dai. Validation: Fangyuan Li, Mengdi Chen. Writing—original draft: Haoran Feng, Junxian Yu. Writing—review & editing: Bingya Liu, Yuan–Yuan Li, Wentao Dai. All authors read and approved the final manuscript. Zhuoqing Xu and Mengdi Chen have accessed and verified the data, and Binya Liu and Yuan–Yuan Li were responsible for the decision to submit the manuscript.

## Data sharing statement

All data are available in the main text or the Supplementary material. The dataset generated is available in the Gene Expression Omnibus database under data series accession number GSE212900.

## Declaration of interests

The authors declare that they have no competing interests.

## Appendix ASupplementary data

Supplementary Figs. S1–S6 and Table S1

Supplemental Western blots

Demographic information of patients
