# Supplementary material for: SLC7A9 suppression increases chemosensitivity by inducing ferroptosis via the inhibition of cystine transport in gastric cancer
Source: eBioMedicine. 2024 Oct 21;109:105375. doi: 10.1016/j.ebiom.2024.105375 (PMC11536348; doi:10.1016/j.ebiom.2024.105375)
Supplement: Supplementary Figs. S1–S6 and Table S1 [file mmc1.docx]

Supplementary figures and tables:

**
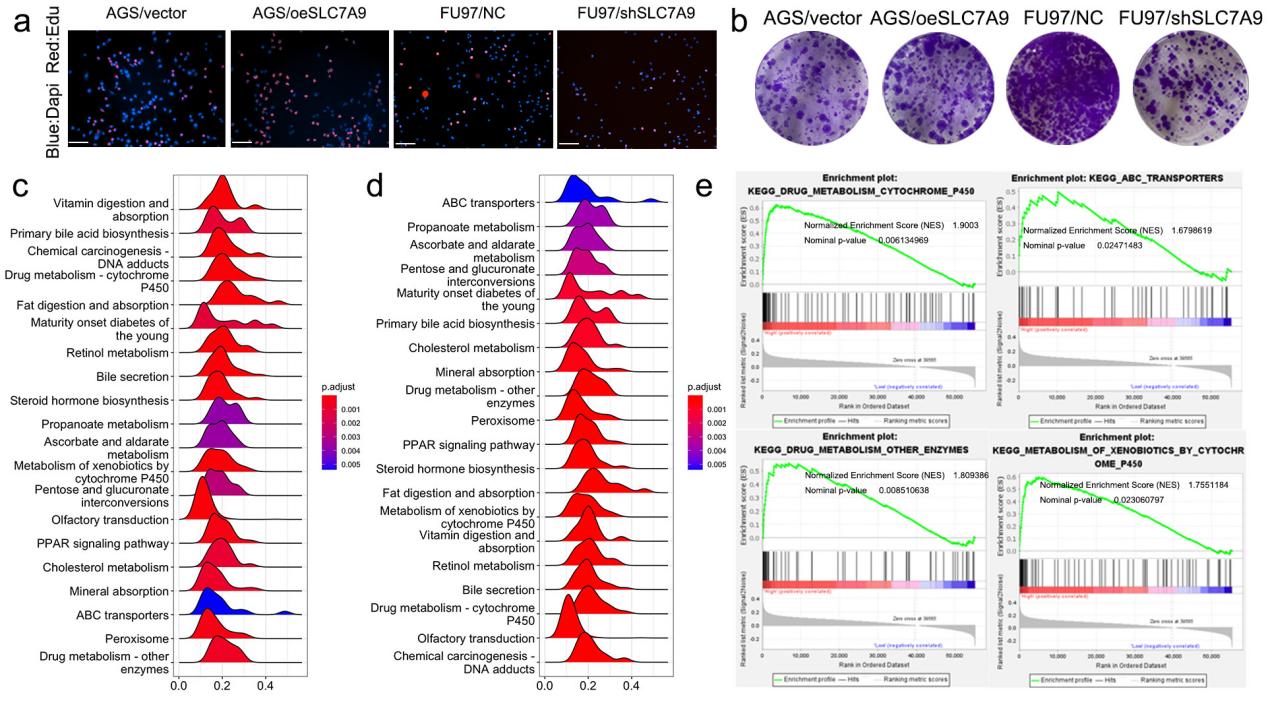
Figure S1 SLC7A9 promoted cell proliferation and correlated with series of pathways regulating drug metabolism.**

**a)** EdU incorporation, scale bar 200μm. **b)** Colony formation assay. **c)** The top 20 GSEA enriched pathways based on SLC7A9 expression in TCGA-STAD database in the order of enrichment score. **d)** The top 20 GSEA enriched pathways based on SLC7A9 expression in the order of p-value. For panels a and b, the x-axis represents log10(NES) values of each pathway. The ridge plot was colored by p-adjusted values. **e)** GSEA enrichment plot of indicated pathways. Fisher’s exact test.

**
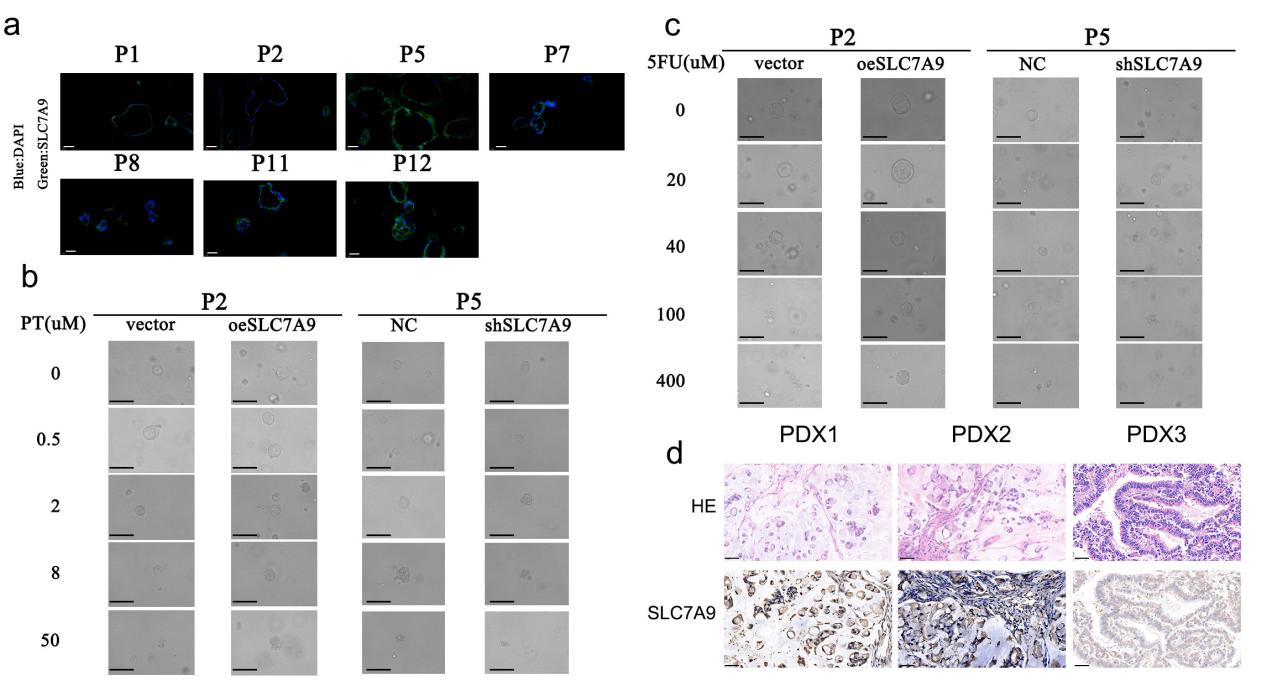
Figure S2 SLC7A9 expressed differently in PDO and PDX and promoted chemoresistance in PDO models of gastric cancer.**

**a)** SLC7A9 expression of 7 PDOs with Immunofluorescence staining, scale bar 40μm. **b)** Representative figures of indicated organoids treated with PT for 4 days, scale bar 100μm. **c)** Representative figures of indicated organoids treated with 5-FU for 4 days. scale bar 100μm. **d)** Representative HE and IHC of indicated PDX. scale bar 40μm.


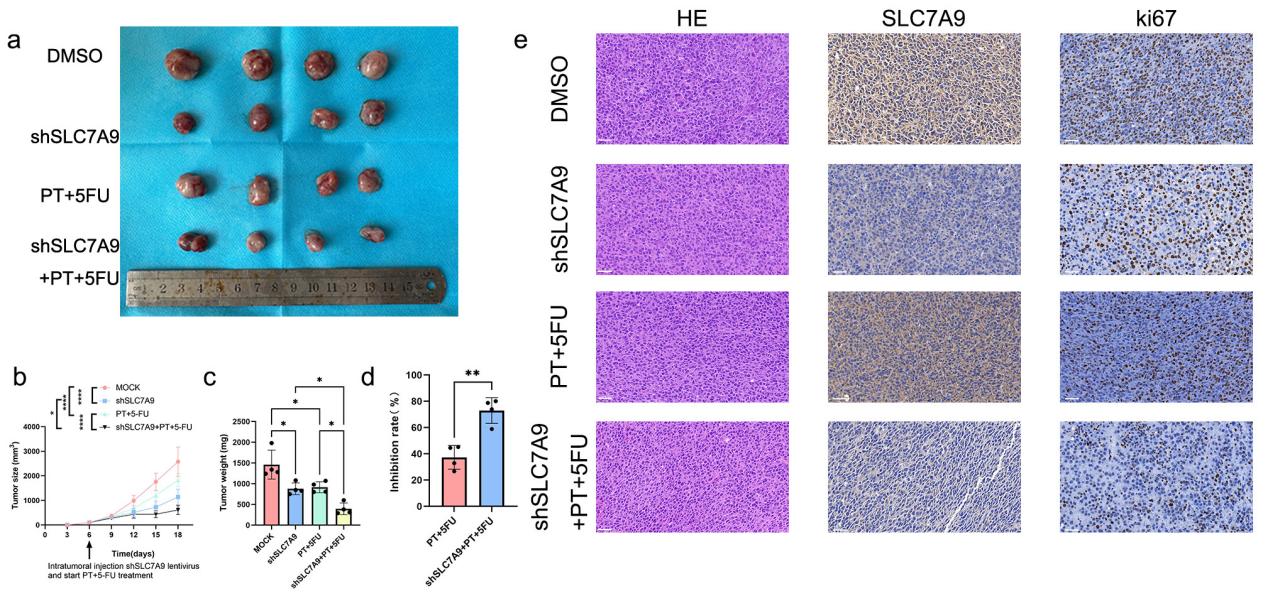


**Figure S3 SLC7A9 enhanced tumour proliferation and tumour resistance to chemotherapy *in vivo*.**

**a)** Tumours from mice with MFC cells subcutaneously injected into 615 mice and treated with shSLC7A9, PT+5-FU or both. **b)** Tumour volumes recorded every 3 days. Two-way ANOVA. **c)** Tumour weight assay. One-way ANOVA. **d)** Tumour inhibition rate of indicate group. Student’s t-tests. **e)** Representative images of HE and IHC staining of ki67 and SLC7A9 on serial sections of tumours from various groups, scale bar 40μm. **p* < 0.05, ***p* < 0.01, *****p* < 0.0001.


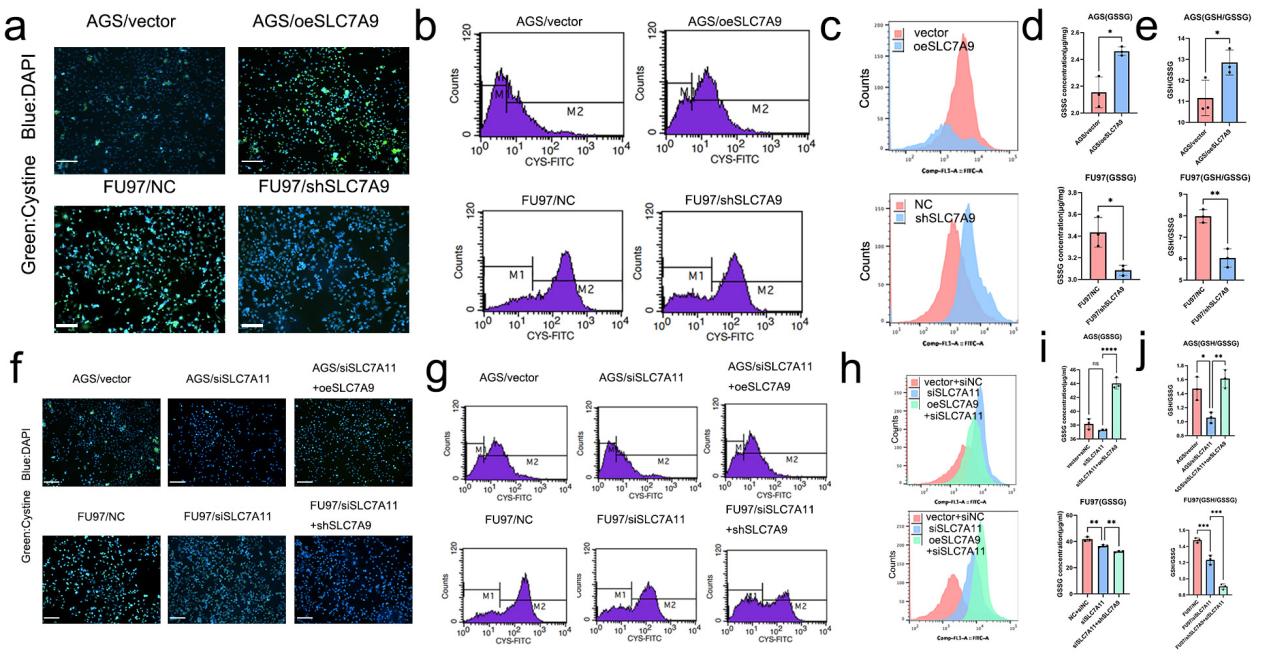


**Figure S4 SLC7A9 promoted cystine intake into cells and enhanced antioxidant ability, independent of SLC7A11.**

**a)** Cystine-FITC fluorescence, scale bar 200μm. **b)** Cell cytometry of cystine-FITC positive cells. M1 represents cells without uptake of cystine-FITC and M2 represents cells with uptake of cystine-FITC. **c)** Lipid-ROS mean fluorescence intensity in each group. ROS probe: C11-BODIPY. **d)** GSSG concentration of indicated groups (μg/mg). Student’s t-tests. e. GSH/GSSG ratio of indicated groups. Student’s t-tests. **f)** Cystine-FITC fluorescence, scale bar 200μm. **g)** Cell cytometry of cystine-FITC positive cells. M1 represents cells without uptake of cystine-FITC and M2 represents cells with uptake of cystine-FITC. **h)** Lipid-ROS mean fluorescence intensity in each group. ROS probe: C11-BODIPY. **i)** GSSG concentration of indicated groups (μg/ml). One-way ANOVA. **j)** GSH/GSSG ratio of indicated groups. One-way ANOVA. ns, not significant, **p* < 0.05, ***p* < 0.01, ****p* < 0.001, *****p* < 0.0001.


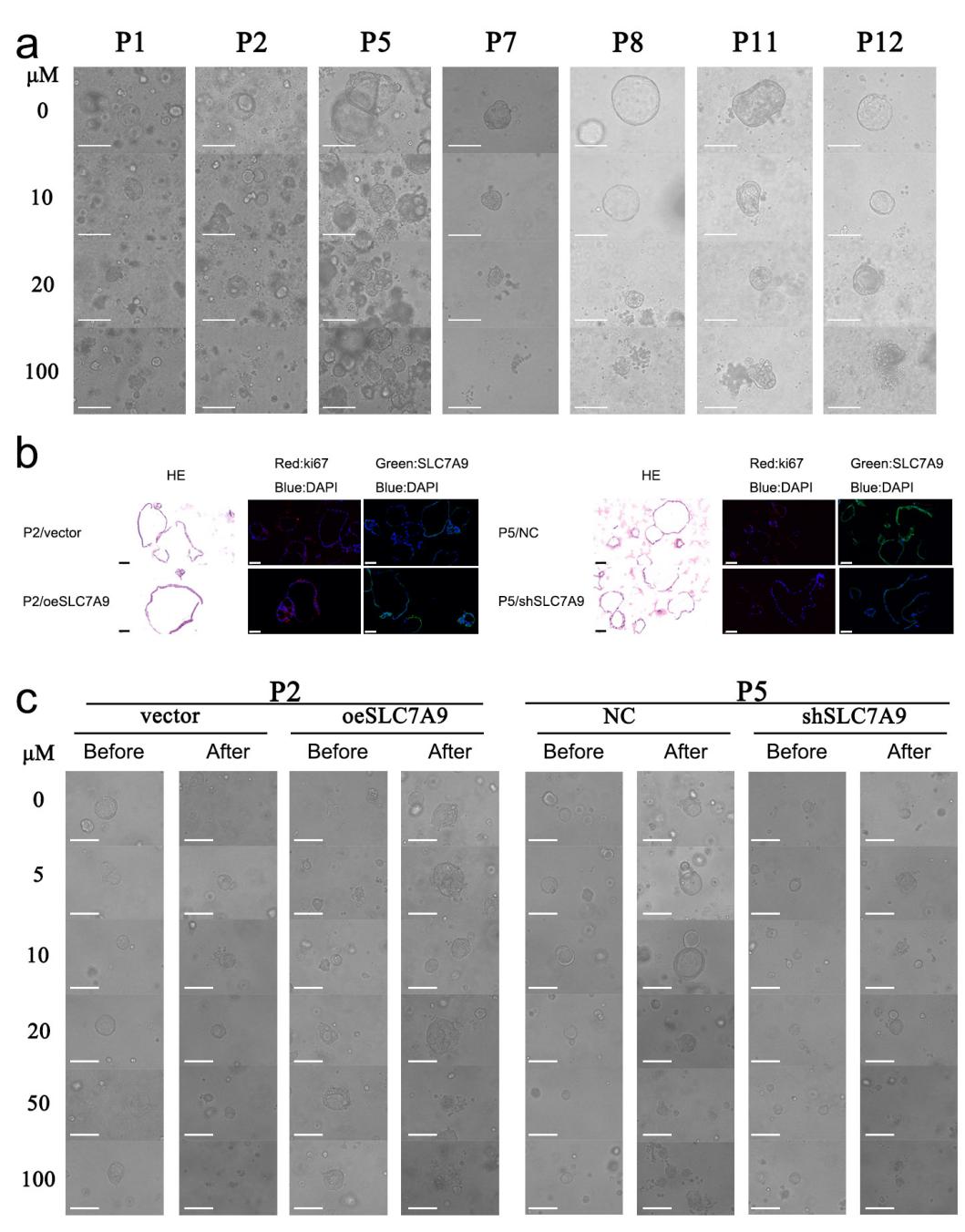


**Figure S5 SLC7A9 resisted to ferroptosis induced by erastin in PDO models of gastric cancer.**

a. Representative figures of 7 PDOs treated without or with erastin 10,20,100 μM for 72h, scale bar 100μm. b. Representative images of HE and Immunofluorescence staining of ki67 and SLC7A9 of tumours from various groups, scale bar 40μm. c. Representative figures of indicated organoids treated without or with erastin 5,10,20,50,100 μM for 72h, scale bar 100μm.


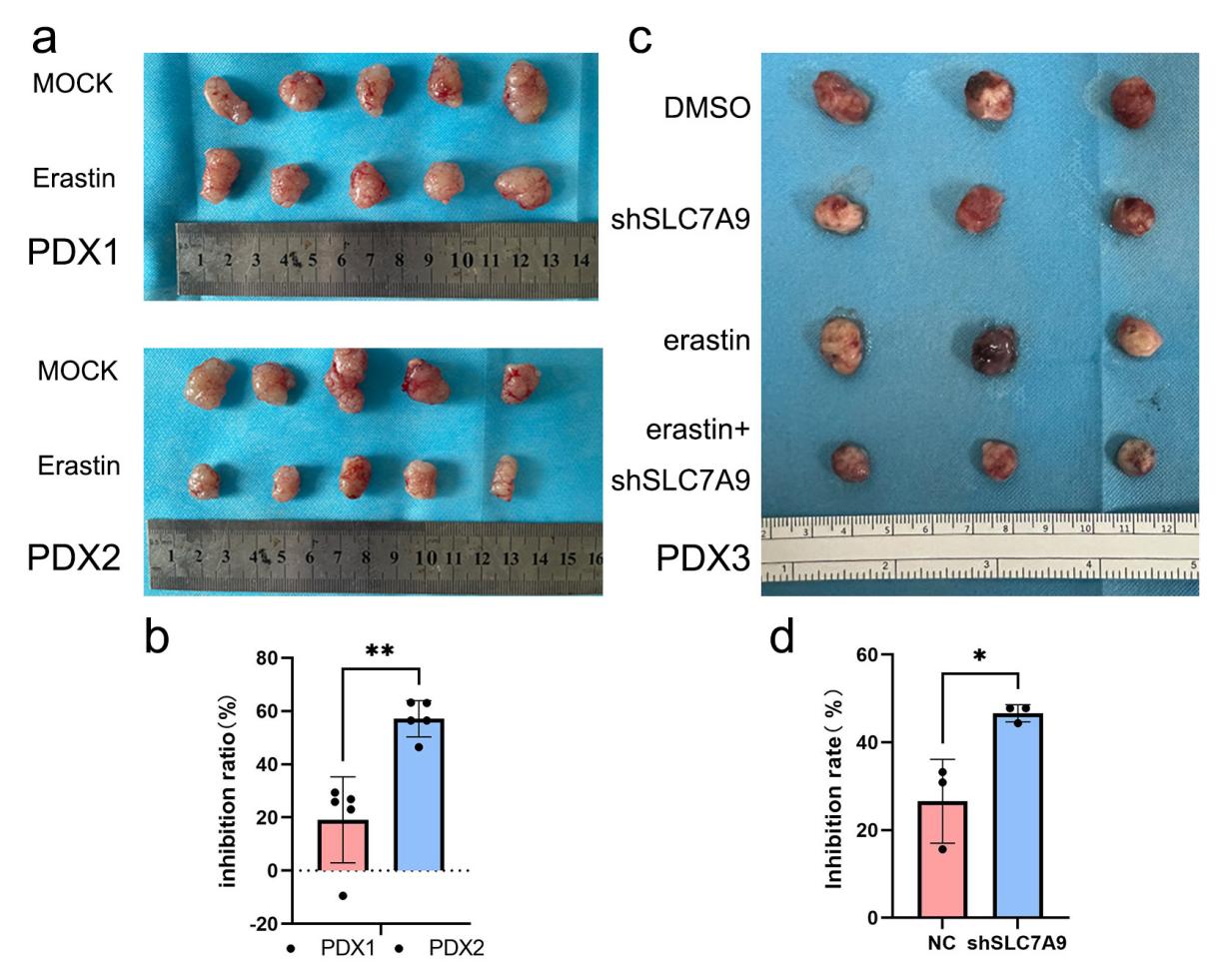


**Figure S6 SLC7A9 mediated tumour resistance to erastin and shSLC7A9 sensitized erastin *in vivo*.**

a. Representative images of PDX tumours. b. Tumour inhibition ratio induced by erastin in 2 PDXs. Student’s t-test. c. Representative images of PDX tumours. d. Tumour inhibition rate of erastin between the two groups. Student’s t-test. **p* < 0.05, ***p* < 0.01.

| Detected area | Direction | Primer sequence |
| --- | --- | --- |
| EXON1 | F： | GCACCCTCCTCCCCAACT |
|  | R： | GCTTCCATCCCACTCACCC |
| EXON2/3/4 | F： | GCTCCACAGGAAGCCGAGCTGTC |
|  | R： | TCAAAAGCCAAGGAATACACG |
| EXON5/6 | F： | GTTTCTTTGCTGCCGTCTTC |
|  | R： | CAAATAAGCAGCAGGAGAAAGC |
| EXON7 | F： | CTGGGCGACAGAGCGAGAT |
|  | R： | GTATGGAAGAAATCGGTAAGAGG |
| EXON8/9 | F： | GGGTGGTTGGGAGTAGATGG |
|  | R： | TGTATCAGGCAAAGTCATAGAACC |
| EXON10 | F： | ACCCCAGAGGCGGAGATT |
|  | R： | GGGTTTGGATGTTCTGTGGATA |
| EXON11 | F： | AAAGCATTGGTCAGGGAAAA |
|  | R： | ACCCCTGCACCTGCTGAC |

Table. S1 Sequence primers of TP53 for sanger sequencing
