# Supplementary figures and images for: SLC7A9 suppression increases chemosensitivity by inducing ferroptosis via the inhibition of cystine transport in gastric cancer
Source: eBioMedicine. 2024 Oct 21;109:105375. doi: 10.1016/j.ebiom.2024.105375 (PMC11536348; doi:10.1016/j.ebiom.2024.105375)

Figure 2d

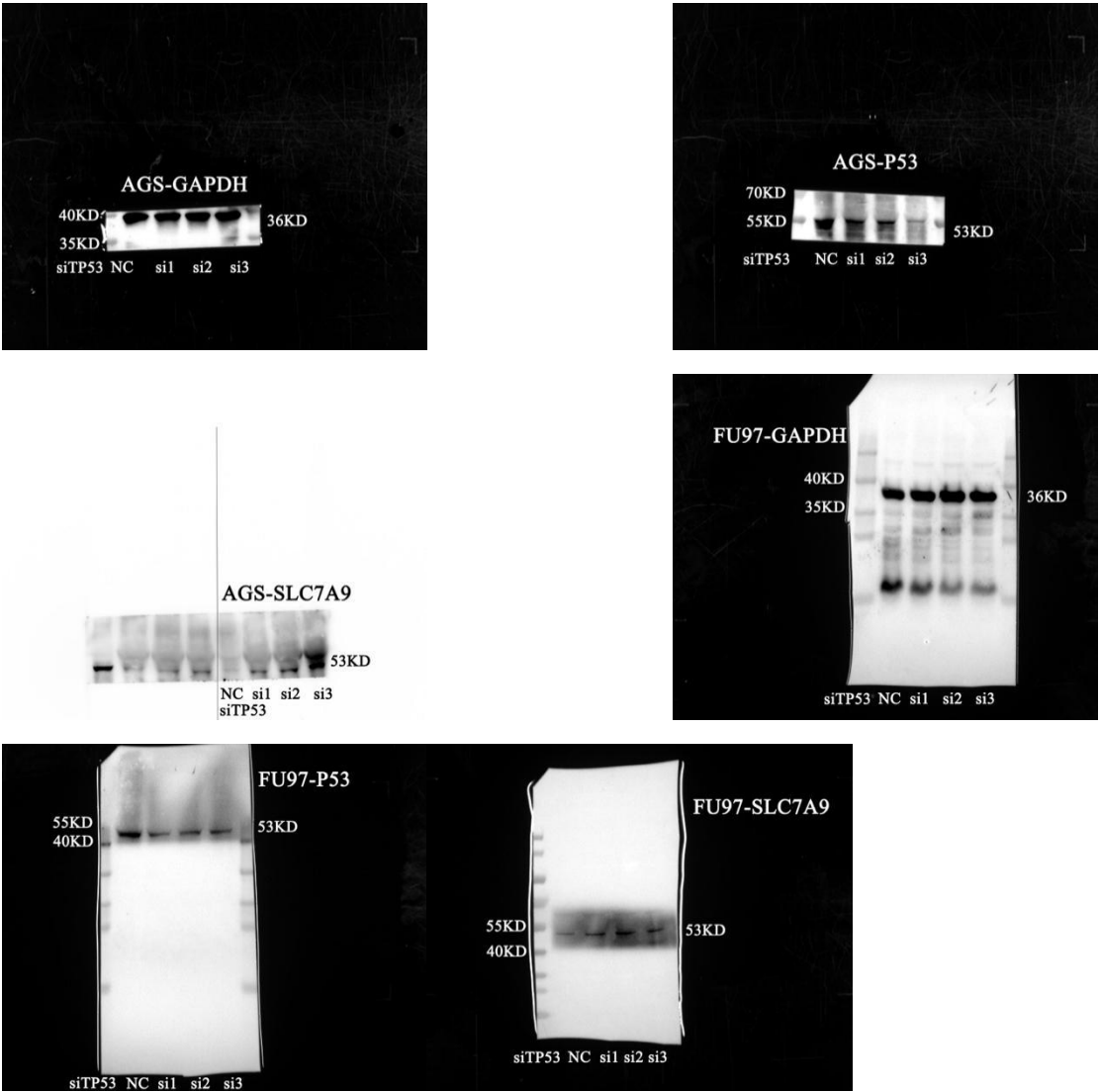

Figure 3a

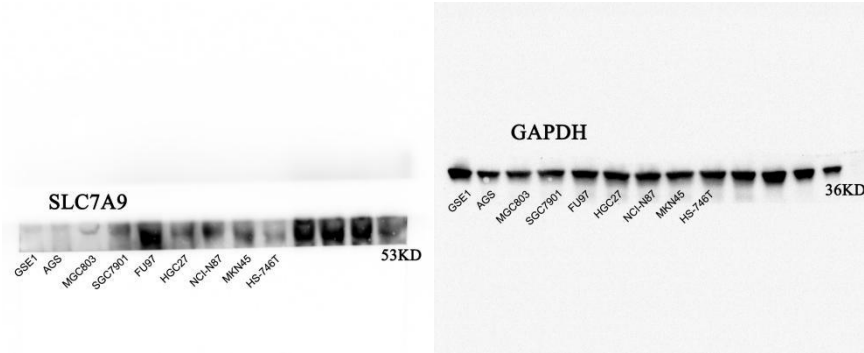

Figure 3b

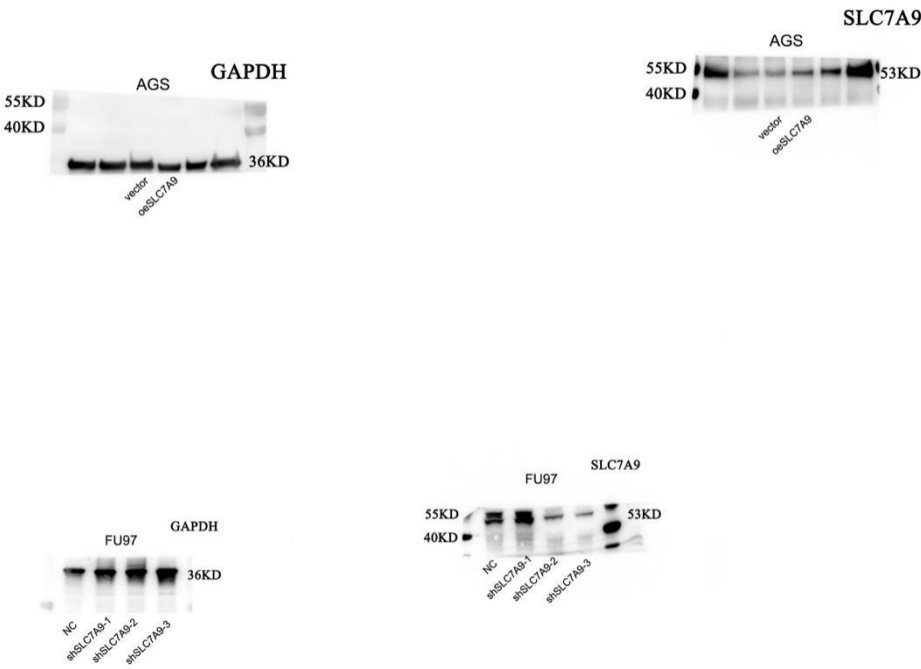

Figure 5i

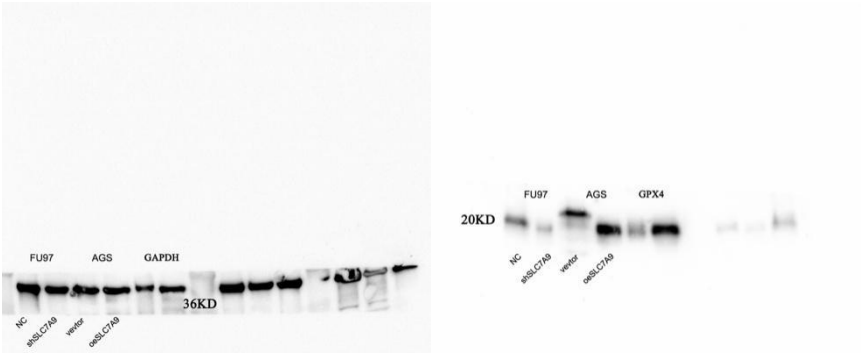

Figure 6a

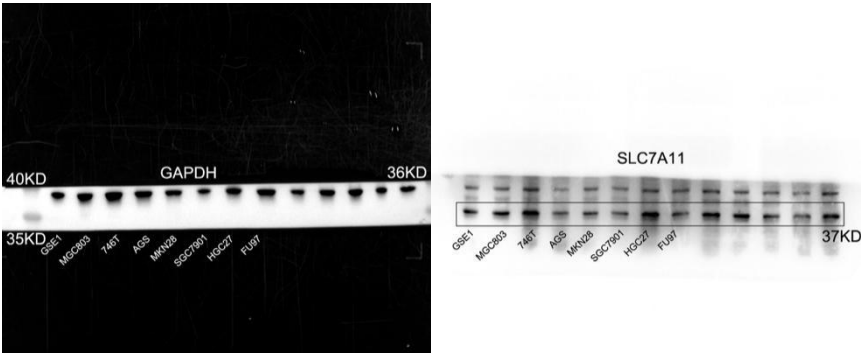

Figure 6b

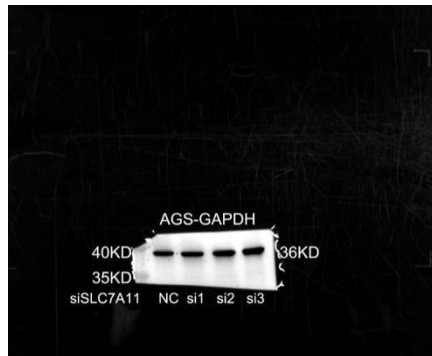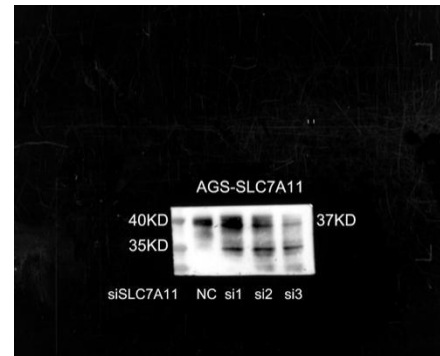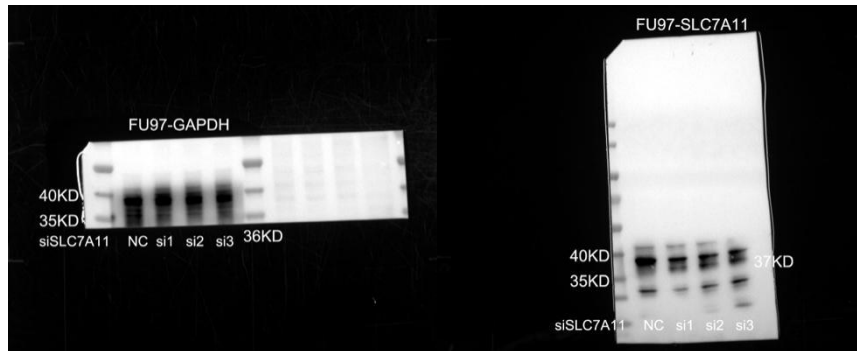

**Figure 6j**

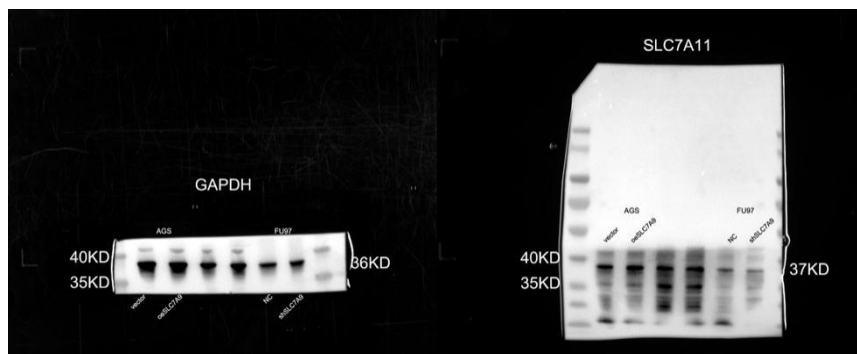

Supplement: Supplemental Western blots [file mmc2.pdf]
