# Supplementary material for: SLC7A9 suppression increases chemosensitivity by inducing ferroptosis via the inhibition of cystine transport in gastric cancer
Source: eBioMedicine. 2024 Oct 21;109:105375. doi: 10.1016/j.ebiom.2024.105375 (PMC11536348; doi:10.1016/j.ebiom.2024.105375)
Supplement: Demographic information of patients [file mmc3.docx]

| **ID** | **Sex** | **Age** | **Local invasion** | **Lymph node metastasis** | **Distant metastasis** | **Pathological type** |
| --- | --- | --- | --- | --- | --- | --- |
| PDO1 | Male | 67 | T4 | N1 | M0 | Adenocarcinoma |
| PDO2 | Female | 63 | T3 | N1 | M0 | Adenocarcinoma |
| PDO5 | Male | 39 | T3 | N3 | M0 | Adenocarcinoma |
| PDO7 | Male | 67 | T2 | N1 | M0 | Adenocarcinoma |
| PDO8 | Male | 53 | T3 | N1 | M0 | Adenocarcinoma |
| PDO11 | Male | 61 | T3 | N1 | M0 | Adenocarcinoma |
| PDO12 | Male | 68 | T3 | N3 | M0 | Adenocarcinoma |
| PDX1 | Female | 48 | T4 | N3 | M0 | signet-ring cell carcinoma |
| PDX2 | Female | 71 | T3 | N1 | M0 | signet-ring cell carcinoma |
| PDX3 | Male | 80 | T4 | N0 | M0 | Adenocarcinoma |
